# AI Trustworthiness in Manufacturing: Challenges, Toolkits, and the Path to Industry 5.0

**DOI:** 10.3390/s25144357

**Published:** 2025-07-11

**Authors:** M. Nadeem Ahangar, Z. A. Farhat, Aparajithan Sivanathan

**Affiliations:** AMRC North West, University of Sheffield, Blackburn BB2 7HP, UK; z.farhat@amrc.co.uk (Z.A.F.); a.sivanathan@amrc.co.uk (A.S.)

**Keywords:** Artificial Intelligence (AI), manufacturing, Industry 4.0, Industry 5.0, AI trustworthiness, transparency, fairness, robustness, accountability, ethical AI, bias mitigation, explainability, AI Toolkits, sustainable manufacturing, human-centric AI

## Abstract

The integration of Artificial Intelligence (AI) into manufacturing is transforming the industry by advancing predictive maintenance, quality control, and supply chain optimisation, while also driving the shift from Industry 4.0 towards a more human-centric and sustainable vision. This emerging paradigm, known as Industry 5.0, emphasises resilience, ethical innovation, and the symbiosis between humans and intelligent systems, with AI playing a central enabling role. However, challenges such as the “black box” nature of AI models, data biases, ethical concerns, and the lack of robust frameworks for trustworthiness hinder its widespread adoption. This paper provides a comprehensive survey of AI trustworthiness in the manufacturing industry, examining the evolution of industrial paradigms, identifying key barriers to AI adoption, and examining principles such as transparency, fairness, robustness, and accountability. It offers a detailed summary of existing toolkits and methodologies for explainability, bias mitigation, and robustness, which are essential for fostering trust in AI systems. Additionally, this paper examines challenges throughout the AI pipeline, from data collection to model deployment, and concludes with recommendations and research questions aimed at addressing these issues. By offering actionable insights, this study aims to guide researchers, practitioners, and policymakers in developing ethical and reliable AI systems that align with the principles of Industry 5.0, ensuring both technological advancement and societal value.

## 1. Introduction

The exponential growth of digital systems in recent years has led to the generation of large-scale, high-dimensional data. This trend is particularly evident in modern manufacturing, where a key shift has been the adoption of decentralised and distributed architectures. In such systems, control and decision-making responsibilities are shared across multiple autonomous units, rather than being managed centrally. This decentralised structure enhances flexibility and resilience, enabling factories to respond swiftly to disruptions and sustain operations despite localised failures. While this distributed approach is often presented as a technical improvement, it also raises critical questions about coordination, accountability, and the reliability of decision-making across autonomous units—issues that are central to the trustworthiness of digital manufacturing systems. Moreover, the distribution of control supports scalable and adaptable production models suited to high variability in demand and complexity [1].

However, this structural evolution also introduces significant challenges. The proliferation of interconnected devices, sensors, and machines leads to massive volumes of heterogeneous data being generated in real time. Traditional analytical tools and human-led analysis are increasingly inadequate for extracting actionable insights from this data deluge. As a result, more advanced, automated, and context-aware data processing methods are required to support intelligent decision-making in distributed manufacturing environment. As a result, Artificial Intelligence (AI) has become essential for intelligent data acquisition, management, and processing [2]. AI enables organisations to analyse large datasets, extract meaningful insights, and support informed decision-making. Efficient data management, supported by AI, not only enhances scalability, security, and operational efficiency but also minimises resource consumption [3]. In this review, we use the term AI to refer specifically to computational systems that can perform tasks typically requiring human intelligence, with a particular emphasis on learning from data and making decisions in complex environments [4]. The concept of knowledge in AI can be categorised into four types: definitional (explicit definitions and facts), deductive (logical inference from rules), inductive (generalisation from examples), and creative (generation of novel ideas) [5]. In this study, we focus on AI as knowledge derived from complex induction, encompassing machine learning, deep learning, and related data-driven approaches. This scope does not include all possible forms of AI, such as purely rule-based or symbolic systems, and is limited to the opportunities and challenges of data-driven, inductive AI in manufacturing environments.

To address the analytical challenges posed by decentralised and data-intensive manufacturing systems, AI and related digital technologies are increasingly employed to create high-fidelity digital models—often referred to as digital twins—that simulate real-world operations. The adoption of digital twins and context-aware processing is not merely a matter of technological advancement; their effectiveness and acceptance depend fundamentally on the trust stakeholders place in the underlying AI systems. Without explicit mechanisms for transparency, fairness, and accountability, these advanced tools risk introducing new vulnerabilities or amplifying existing biases. These models enable manufacturers to evaluate decision scenarios in a virtual environment, assess the impact of process changes, and prioritise risk mitigation strategies. By proactively identifying and responding to operational challenges, factories can improve resilience, reduce economic disruptions, and seize emerging opportunities with greater agility.

Building on the foundation established by Industry 4.0, the emerging paradigm of Industry 5.0 places greater emphasis on human-centric approaches, sustainability, and ethical considerations. In this new era, AI continues to play a pivotal role—not only in driving automation and efficiency but also in supporting more responsible and inclusive industrial practices [6,7]. To further conceptualise the flow and use of information in Industry 4.0 and 5.0, it is helpful to consider three fundamental components: Syntax, Semantics, and Pragmatics. Syntax refers to the structure and format of data, ensuring interoperability between systems. Semantics addresses the meaning and interpretation of data, enabling both machines and humans to derive actionable insights. Pragmatics, however, concerns the practical application and real-world impact of information—how data-driven outputs are used in operational contexts. Critically, trustworthiness is a central aspect of Pragmatics, as it determines whether stakeholders can reliably act on AI-generated insights in manufacturing environments. This perspective highlights that trustworthiness is not merely a technical attribute but a practical necessity for the successful and responsible adoption of AI in Industry 4.0/5.0 [8]. However, the adoption of AI in manufacturing is not without significant challenges. One major concern is the “black box” nature of many AI models, which refers to the difficulty in understanding how these systems arrive at their decisions or predictions. This lack of transparency and interpretability can hinder trust and accountability, as stakeholders may be unable to trace or justify the reasoning behind AI-driven outcomes [9,10].

Trust in AI, particularly in high-stake industrial contexts, is a multidimensional construct. Drawing from the broader trust literature, trust can be understood as comprising three interrelated components: scientific or technical competence, effective communication, and shared values [11]. While much of the AI literature focuses on technical robustness and explainability (science/competence), empirical research consistently finds that failures of trust are more often rooted in value misalignments and poor communication than in technical shortcomings. As Greenberg [11] notes, value-based trust is often the most challenging to build and maintain, especially when organisational or societal values are perceived to be at odds with those of affected stakeholders.

Another critical issue involves biases present in both the data used to train AI systems and the algorithms themselves. Biases can arise from historical data that reflect existing inequalities or from the design of algorithms that inadvertently favour certain groups or outcomes over others. In manufacturing, such biases may result in unfair resource allocation, exclusion of certain workforce segments, or suboptimal decision-making that does not account for the diversity of real-world scenarios. These concerns threaten the fairness and inclusivity of AI applications, making it essential to identify, measure, and mitigate bias throughout the AI lifecycle [9].

In this study, AI trustworthiness is defined as the degree to which AI systems can be relied upon to operate transparently, fairly, robustly, and accountably within manufacturing environments. Drawing on established frameworks such as the European Commission’s Ethics Guidelines for Trustworthy AI, we operationalise AI trustworthiness through its core dimensions. Transparency refers to the extent to which AI decision-making processes are understandable and explainable to stakeholders, enabling traceability and auditability. Fairness is the assurance that AI systems do not propagate or amplify bias and that outcomes are equitable across different groups and contexts. Robustness signifies the resilience of AI systems to errors, adversarial attacks, and changing operational conditions, ensuring reliable performance. Accountability denotes the presence of mechanisms for assigning responsibility and enabling recourse in the event of system failures or unintended consequences. In the manufacturing domain, these dimensions are particularly salient due to the high stakes associated with safety, quality, and regulatory compliance. Measurable characteristics of AI trustworthiness in this context include the availability of model documentation, bias detection and mitigation reports, robustness testing results, and clear lines of responsibility for AI-driven decisions [12].

The motivation for this paper stems from the urgent need to address these challenges and bridge the gap between rapid technological advancements and their ethical, human-centric application in manufacturing. Furthermore, the dynamic and complex nature of manufacturing environments amplifies the risks associated with AI failures, which can lead to significant supply chain disruptions, reduced business efficiency, loss in production capacity, ethical violations, and loss of stakeholder trust. Despite a growing body of research on AI in manufacturing, there remains a critical gap in comprehensively addressing the trustworthiness of AI systems. This gap is particularly significant in the context of Industry 5.0, where aligning AI technologies with human-centric and sustainable principles is paramount. To address these challenges, a range of organisations and regulatory bodies have established comprehensive frameworks to promote the trustworthy and responsible use of AI. Notable examples include the European Commission’s Ethics Guidelines for Trustworthy AI, the National Institute of Standards and Technology (NIST) AI Risk Management Framework, the Organisation for Economic Co-operation and Development (OECD) Principles on Artificial Intelligence, the International Organization for Standardization (ISO) and International Electrotechnical Commission (IEC) standards on AI trustworthiness, the IEEE Ethically Aligned Design, and the Singapore Model AI Governance Framework. These frameworks provide structured approaches for organisations to assess, monitor, and improve the reliability, fairness, and ethical alignment of AI systems [13,14]. However, these frameworks differ in their scope, rigour, and practical enforceability. For example, while the European Commission’s guidelines emphasise ethical principles, the NIST and ISO/IEC standards focus more on technical and procedural aspects. Contradictions and gaps remain, particularly regarding how these frameworks address the unique operational realities of manufacturing, such as real-time decision-making and the integration of legacy systems [15].

Nevertheless, there remains a critical research gap: the absence of comprehensive frameworks and methodologies specifically tailored to the unique demands of manufacturing environments. This paper aims to address this gap by providing a comprehensive survey of AI trustworthiness in manufacturing. The primary objectives of this study are the following:

1. Critically examine the role of AI in the transition from Industry 4.0 to Industry 5.0, with a focus on the technical, ethical, and organisational challenges specific to manufacturing.
2. Assess the effectiveness and limitations of existing toolkits for ensuring AI trustworthiness—specifically transparency, fairness, robustness, and accountability in manufacturing contexts.
3. Formulate targeted research questions and methodological approaches to address the most pressing challenges of AI adoption in manufacturing, drawing on industry case studies

The findings of this study are expected to guide professionals, engineers, and decision-makers in manufacturing to adopt AI in ways that improve processes and respect societal and environmental values.

**Figure 1:**
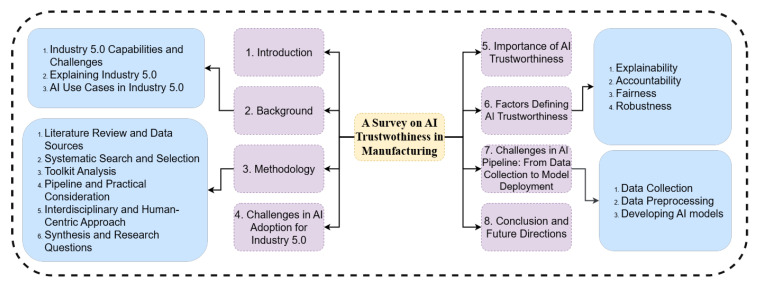
Overall paper structure.

The structure of this paper, illustrated in Figure 1, is organised into eight sections, each addressing a critical dimension of AI trustworthiness in the context of Industry 5.0. Section 2 provides the background, tracing the evolution from Industry 4.0 to Industry 5.0 and emphasising the shift toward human-centricity, sustainability, and ethical integration. It further details the collaborative nature of Industry 5.0 and enumerates practical AI use cases in manufacturing, such as digital twins, predictive maintenance, and generative design. This section sets the stage for addressing the first research question: What is the role of AI in the transition from Industry 4.0 to Industry 5.0, and what are the associated challenges? Section 3 outlines the multi-stage methodology, encompassing a literature review, systematic search and selection, toolkit analysis, and an interdisciplinary, human-centric approach to synthesising research questions. This section establishes the methods used to explore the research questions and address AI adoption challenges in manufacturing. Section 4 examines the challenges in AI adoption for Industry 5.0, including technical, organisational, and ethical barriers like black-box models, data bias, reliability, regulatory concerns, security threats, and workforce adaptation. This section directly addresses the second part of the first research question: What are the technical, ethical, and organisational challenges of AI adoption in manufacturing? Section 5 discusses the importance of AI trustworthiness, drawing on real-world failures to highlight the necessity of transparency, fairness, and accountability in building trust and preventing harm. This section underscores the need for trustworthy AI systems and sets the context for exploring the necessary toolkits. Section 6 defines the core factors of AI trustworthiness: explainability, accountability, fairness, and robustness. It reviews key toolkits and frameworks for each factor, addressing the need for interpretable decisions, responsible governance, context-specific fairness, and resilience against errors and adversarial attacks. This section directly addresses the second research question: What toolkits are necessary to ensure AI trustworthiness, such as transparency, fairness, robustness, and accountability? Section 7 analyses challenges across the AI pipeline, from data collection and preprocessing to model development and deployment, and formulates research questions on data integrity, bias detection, labelling consistency, and the trade-offs between interpretability and performance. This section delves into the methods to address AI adoption challenges in manufacturing, supported by industrial examples, thus addressing the third research question. Section 8 concludes by summarising the progress in trustworthy AI frameworks and toolkits, underscoring the ongoing need for ethical, technical, and organisational vigilance. It calls for interdisciplinary collaboration, regulatory compliance, and practical evaluation of toolkits in real-world scenarios and outlines future research directions to ensure continuous monitoring and adaptation of AI systems in manufacturing. This section synthesises the findings and provides a roadmap for future research, addressing all three research questions.

## 2. Background

### 2.1. Industry 5.0 Capabilities and Challenges

The evolution of modern manufacturing began with Industry 4.0, which is characterised by the integration of cyber–physical systems, the Internet of Things (IoT), and advanced data analytics into industrial processes. Industry 4.0 has enabled unprecedented levels of automation, connectivity, and data-driven decision-making, transforming traditional factories into smart, interconnected environments. However, this transformation has also introduced significant challenges, such as managing the complexity of large-scale data, ensuring cybersecurity, and addressing the skills gap required to operate and maintain advanced technologies.

**Figure 2:**
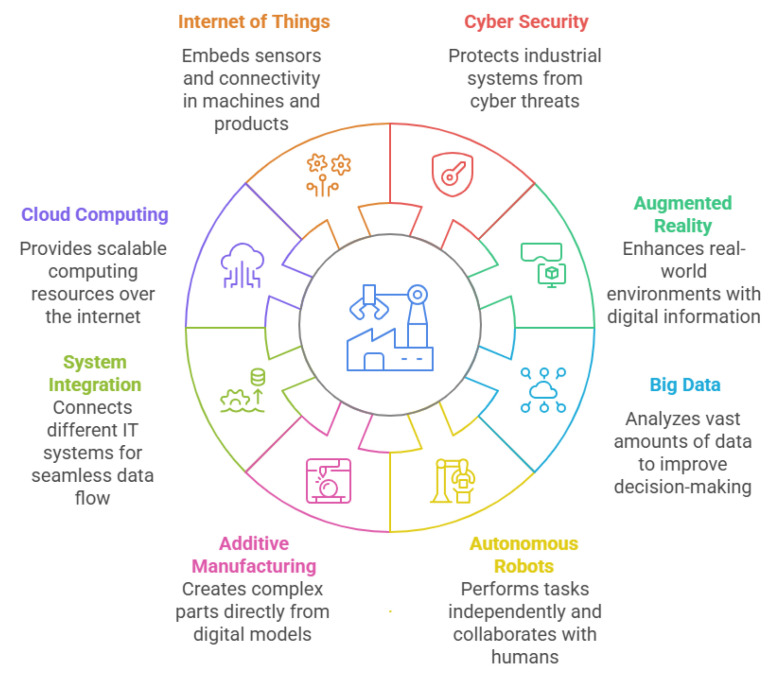
Key technologies in Industry 4.0.

Figure 2 outlines the key pillars driving Industry 4.0, as identified in [16]. The influence of these technologies now extends well beyond traditional industrial settings, shaping home products, business models, clean energy solutions, and broader sustainability efforts—areas that earlier industrial revolutions largely overlooked. As a result, industry is increasingly recognised as a catalyst for systemic transformation, pushing economies toward greater sustainability [17]. Achieving this shift requires the integration of societal and environmental considerations as core priorities within the industrial sector.

This move toward decentralisation has been made possible by the widespread adoption of sensors and actuators embedded in machines through the IoT. These devices create seamless connectivity with computing systems and generate vast streams of data, commonly referred to as Big Data [18]. To handle this data efficiently, processing often occurs locally on IoT devices or is distributed through cloud and edge computing platforms. This approach not only optimises costs and improves scalability by leveraging virtual resources [19] but also supports the adoption of new technologies aligned with Industry 4.0 objectives. AI technologies are uniquely capable of rapidly collecting and analysing information from multiple systems, enabling tasks such as fault prediction and action selection to be performed with far greater efficiency [20]. Consequently, many companies are adopting intelligent systems that support various levels of process automation, further accelerating the transformation of the manufacturing industry and supporting the broader goals of Industry 4.0 and beyond.

The European Commission introduced the concept of Industry 5.0 in 2020 during a dedicated workshop involving research and technology organisations and funding bodies. This new paradigm integrates AI and the societal dimension as key drivers for the future of European industry [21]. Since then, multiple initiatives have been launched to support Industry 5.0, including efforts to upskill and reskill European workers, particularly in digital competencies (Skills Agenda and Digital Education Action Plan); fostering a more competitive industrial landscape through accelerated investment in research and innovation (Industrial Strategy); promoting sustainable development through resource-efficient, eco-friendly industries and a transition to a circular economy (Green Deal); and advocating for a human-centric approach to digital technologies via regulatory frameworks such as the AI Act, white papers, and trustworthy AI requirements [22,23,24,25,26,27].

A central pillar of Industry 5.0 is AI adoption, with a focus on high-speed data processing, workforce expertise in managing AI-driven heterogeneous technologies (including computing resources and data), and embedding ethical principles throughout the AI lifecycle to ensure trust and safe working environments [28,29]. While Industry 5.0 aims to foster collaboration between humans and machines and promote ethical, sustainable industrial practices, it also faces its own set of challenges. These include integrating ethical principles into AI systems, ensuring workforce adaptability, and balancing technological progress with societal and environmental considerations. While Industry 5.0 is often presented as a progressive and human-centric evolution of manufacturing, several of its core assumptions warrant critical examination. For example, the notion that increased human–machine collaboration will automatically lead to more ethical or sustainable outcomes is not universally supported by empirical evidence. There is ongoing debate about whether the integration of advanced AI and automation truly empowers workers or, conversely, risks further deskilling and job displacement. Additionally, the emphasis on sustainability and resilience in Industry 5.0 frameworks can sometimes mask the persistent tension between economic growth and environmental limits, raising questions about the feasibility of achieving all three goals simultaneously. Critics also point out that the practical implementation of ethical AI principles remains challenging, with many organisations struggling to translate high-level values into operational practices. As such, while Industry 5.0 offers an aspirational vision, its real-world impact depends on addressing these unresolved tensions and ensuring that technological progress is matched by genuine social and environmental responsibility [7].

Despite ongoing research efforts to integrate ethical considerations into AI applications, unique challenges persist depending on the operational environment and the specific domains where these technologies are deployed [30].

### 2.2. Explaining Industry 5.0

In the context of Industry 5.0, the connection between factory-specific challenges, AI applications, and technological pillars remains insufficiently defined. Industry 5.0 marks a fundamental shift that extends beyond technological and economic aspects, placing a strong emphasis on human well-being, sustainability, and circular economies. Unlike previous industrial advancements that focused on automation and efficiency, Industry 5.0 promotes a collaborative relationship between humans and machines, leveraging their unique strengths rather than aiming for human replacement [31]. This paradigm shift calls for a more holistic integration of AI, where ethical considerations and societal impacts are prioritised alongside technical advancements.

While Industry 5.0 aspires to be human-centric and ethical, these ambitions often involve complex trade-offs and can lead to unintended consequences. For example, efforts to enhance worker well-being through increased human–machine collaboration may inadvertently introduce new forms of workplace stress, such as the need for constant upskilling or the psychological impact of working alongside intelligent machines. Similarly, prioritising ethical AI can sometimes slow down innovation or increase operational costs, as organisations must invest in transparency, bias mitigation, and compliance measures. There is also the risk that well-intentioned ethical frameworks may be inconsistently applied, leading to gaps between policy and practice. These examples highlight that the pursuit of human-centric and ethical objectives in Industry 5.0 is not without challenges, and careful consideration of potential trade-offs is essential for responsible implementation [32].

In addition to these user-facing applications, AI is increasingly being deployed within the underlying infrastructure, such as edge computing. Here, AI enables real-time data processing for tasks like predictive maintenance and supports advanced features, including Augmented Reality experiences. This seamless integration of AI across both consumer applications and technical infrastructure demonstrates its versatility and growing importance in modern technology ecosystems [33]. As AI continues to evolve, its role in shaping both the digital landscape and industrial environments will become even more significant, underscoring the need for ongoing research and thoughtful implementation.

**Figure 3:**
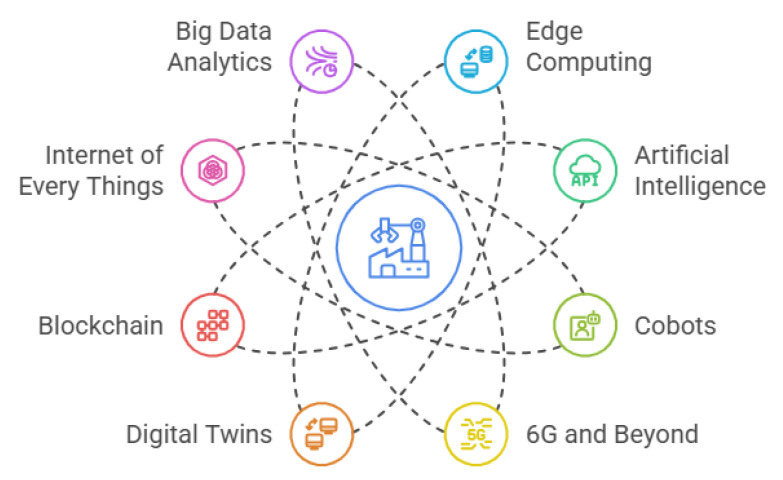
Key technologies in Industry 5.0.

Several key technologies serve as enablers of Industry 5.0, as identified by authors in Xu and Duan [2], Xu et al. [7], Commission et al. [21], Wang et al. [31], Habib ur Rehman et al. [34], Vyhmeister et al. [35], Vyhmeister et al. [36], Wang et al. [37], Wu et al. [38]. These enablers, outlined in Figure 3, represent core components that drive this new industrial paradigm [39].

Future industries are expected to play an important role in advancing societal goals while contributing to a more environmental friendly and sustainable ecosystem [40].

### 2.3. AI Use Cases in Industry 5.0

**Figure 4:**
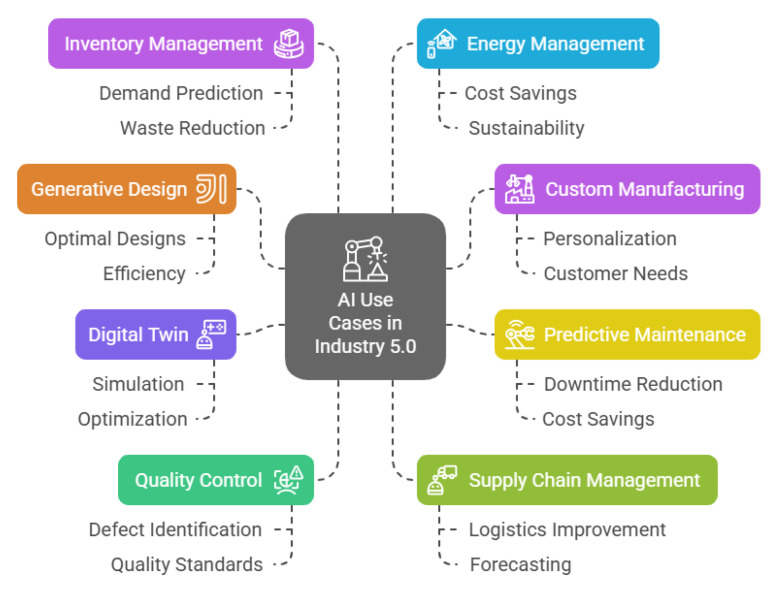
AI use cases in Industry 5.0.

AI plays a significant role in Industry 5.0 by enabling smarter, more efficient, and adaptable operations. The following examples shown in Figure 4 illustrate AI’s potential applications within the Industry 5.0 framework [16,41,42]:

1. Digital Twin: AI is utilised to create virtual representations of processes, production systems, factories, and supply chains, referred to as digital twins. These virtual models are employed to simulate, evaluate, and predict performance in real-time. By replicating the physical environment, digital twins allow manufacturers to monitor and improve operations without needing direct engagement with the physical assets. They depend on data from IoT sensors, programmable logic controllers (PLCs), deep learning techniques, and AI algorithms to continuously update the digital model with real-time information, ensuring an up-to-date and accurate virtual replica.
2. Predictive maintenance: AI processes sensor data from machinery to predict potential failures before they happen. By utilising a digital twin to examine patterns in equipment behaviour and performance, these systems can notify operators of potential issues in advance, enabling them to prevent breakdowns before they worsen. For instance, automotive manufacturers use predictive maintenance on assembly-line robots, greatly decreasing unplanned downtime and leading to significant cost savings. This method also allows manufacturers to schedule maintenance during off-peak hours, minimising disruptions to production timelines [43].
3. Custom Manufacturing: AI empowers manufacturers to provide mass customisation, enabling products to be tailored to individual customer preferences without disrupting production speed. By incorporating AI into the design process, companies can swiftly adjust designs in response to real-time consumer feedback. For example, clothing manufacturers utilise AI algorithms to personalise products, allowing customers to select designs that align with their unique tastes. This adaptability not only improves customer satisfaction but also boosts engagement by offering a more personalised shopping experience.
4. Generative Design: This technology allows manufacturers to explore numerous design possibilities by considering factors like materials and manufacturing limitations. This approach accelerates the design process by enabling the rapid evaluation of multiple iterations. Generative AI design tools are already being utilised in industries like the aerospace and automotive industries, where companies use them to develop optimised parts. Although the technology is already in use, its complete potential is still not being explored within the dynamic landscape of modern manufacturing.
5. Quality Control: AI improves quality control by using computer vision and machine learning, often supported by a digital twin, to detect defects in real-time. These systems examine product images during the manufacturing process, identifying inconsistencies or faults with greater precision than human inspectors. For example, electronic manufacturers utilise AI-driven quality control to ensure components meet stringent specifications. This results in higher product quality, reduced waste, and greater customer satisfaction.
6. Supply Chain Management: AI streamlines supply chain operations by analysing large volumes of data to forecast demand, manage stock levels, and improve logistics. When coupled with a digital twin, AI can build a virtual model of the entire supply chain, enabling manufacturers to predict and simulate disruptions or shortages in real-time. Machine learning assists with demand predictions and automates procurement, ensuring that manufacturers receive materials precisely when needed. AI-driven order management systems also optimise order fulfilment, ensuring deliveries are made on time. For example, food manufacturers use AI to anticipate seasonal shifts in demand, allowing them to better manage resources and reduce waste. This ultimately boosts operational efficiency and enhances responsiveness to market fluctuations.
7. Inventory management: AI enhances inventory management by analysing data to predict stock requirements and streamline replenishment. By forecasting demand and tracking inventory in real-time, manufacturers can ensure optimal stock levels, lowering storage costs and improving cash flow. For instance, food and beverage manufacturers use AI systems to monitor ingredient consumption as it happens. This enables them to predict future needs based on production timelines, seasonal factors, and historical usage, helping to avoid production disruptions and minimise waste from excess stock.
8. Energy Management: AI systems track energy consumption in real-time to pinpoint inefficiencies. These systems can suggest changes that help cut energy costs and reduce environmental impact. For example, electronic manufacturers use AI-driven energy management solutions to improve their operations, leading to substantial cost reductions and a smaller carbon footprint.

While these AI applications offer significant promise, their deployment in manufacturing has also revealed critical challenges related to trust, fairness, and explainability. In manufacturing, “ethical” AI refers to systems that operate transparently, avoid bias, respect stakeholder values, and ensure accountability for outcomes [12]. However, real-world failures highlight the complexity of achieving these goals. Given the critical role of AI in Industry 5.0, industries are increasingly cautious about its adoption due to concerns over transparency, ethical risks, regulatory compliance, and reliability. Without clear governance and accountability, AI adoption remains a challenge, particularly in high-risk sectors.

## 3. Methodology

**Figure 5:**
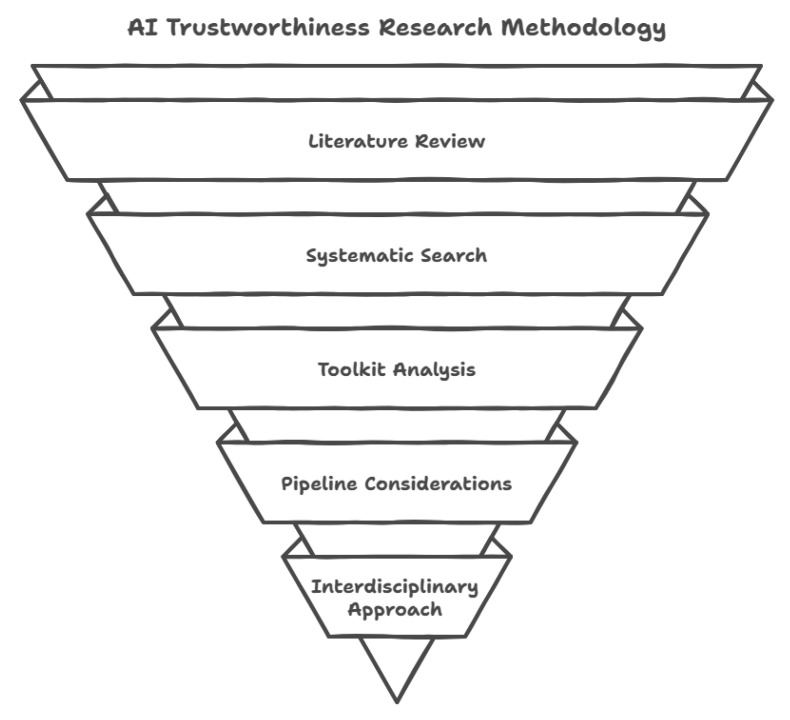
Overall methodology.

This study adopts a rigorous, multi-stage methodology to systematically investigate the challenges and enablers of AI trustworthiness in manufacturing, particularly within the context of evolving industrial paradigms. The approach highlighted in Figure 5 is designed to ensure both breadth and depth, combining a comprehensive literature review, critical analysis of toolkits, and the use of illustrative case studies to provide a holistic understanding of AI trustworthiness.

1. Literature Review and Data Sources: This research begins with an extensive literature review, targeting peer-reviewed journal articles, conference proceedings, and authoritative industry reports. Sources are drawn from high-impact databases including IEEE Xplore, Scopus, Web of Science, and the ACM Digital Library. To ensure relevance and currency, this review is limited to works published within the last decade, with a particular emphasis on studies addressing AI trustworthiness in manufacturing. In addition, regulatory documents and guidelines—such as ISO/IEC standards, the European Union (EU) AI Act, and the Assessment List for Trustworthy Artificial Intelligence (ALTAI)—are included to capture the evolving landscape of ethical and legal requirements.
2. Systematic Search and Selection: A systematic search strategy is employed, using targeted keywords such as “AI trustworthiness”, “Industry 5.0”, “ethical AI”, “transparency in AI”, “Toolkits in AI”, and “manufacturing.” The selection process prioritises studies that address the core dimensions of trustworthy AI—transparency, fairness, robustness, and accountability—within manufacturing contexts. The inclusion of case studies, both of AI successes and failures, provides practical grounding and validation for the findings. To ensure a comprehensive and transparent literature review, the search strategy involved querying databases using specific search strings like “AI trustworthiness” AND “manufacturing”, “AI ethics” AND “smart manufacturing”, and “responsible AI” AND “industry 5.0”. Inclusion criteria were applied to select peer-reviewed articles, conference papers, and relevant reports published between 2015 and 2024, focusing on AI trustworthiness in manufacturing applications. Exclusion criteria were used to filter out studies that were not directly relevant to the manufacturing sector or did not address AI trustworthiness. The initial search yielded 500 of articles, which were then screened based on their titles and abstracts. Full-text reviews were conducted on 300 articles, resulting in a final selection of 200 articles that met the inclusion criteria.
3. Toolkit Analysis: The analysis is structured around four key dimensions of AI trustworthiness: transparency, fairness, robustness, and accountability. For each dimension, this study critically examines a range of prominent toolkits and frameworks, including but not limited to AI Explainability 360 (AIX360), SHapley Additive exPlanations (SHAP), Local Interpretable Model-agnostic Explanations (LIME), AI Fairness 360 (AIF360), FairLearn, IBM Adversarial Robustness Toolbox (IBM ART), and CleverHans. The discussion evaluates the strengths, limitations, and practical applications of these tools, offering a comprehensive perspective on their contributions to trustworthy AI in manufacturing. The evaluation criteria included: (1) transparency mechanisms (e.g., explainable AI (XAI) techniques), (2) fairness metrics and mitigation strategies, (3) robustness testing and validation methods, (4) accountability frameworks, and (5) ethical guidelines and compliance support. The toolkits were assessed based on their functionalities, ease of use, and applicability to manufacturing contexts. The evaluation involved a qualitative, comparative analysis, drawing upon expert judgment to assess the toolkits’ strengths and weaknesses in addressing AI trustworthiness concerns. A formal numerical scoring system was not used due to the diversity of toolkit functionalities and the context-dependent nature of manufacturing applications. Instead, the evaluation focused on providing a nuanced understanding of each toolkit’s capabilities and limitations in promoting AI trustworthiness.
4. Pipeline and Practical Considerations: The methodology explicitly addresses challenges across the entire AI pipeline—from data collection and preprocessing to model training, deployment, and post-deployment monitoring. Special attention is given to issues such as data quality, interoperability, bias, concept drift, and the integration of domain expertise.
5. Interdisciplinary and Human-Centric Approach: Recognising the complexity of manufacturing environments, the methodology emphasises interdisciplinary collaboration among AI developers, domain experts, and end-users. This ensures that technical solutions are both practically relevant and ethically aligned. The approach is further informed by the human-centric and sustainable ethos of Industry 5.0, integrating ethical considerations and stakeholder perspectives at every stage. No new stakeholder interviews or primary qualitative data were collected; instead, the human-centric perspective is embedded through the integration of ethical considerations and stakeholder insights from published qualitative studies and documented experiences. To illustrate the potential challenges and implications of AI trustworthiness in manufacturing, this study employs a series of hypothetical case studies. These scenarios are not based on specific real-world implementations but are carefully constructed to represent common AI applications across diverse manufacturing sectors such as automotive, aerospace, and electronics. The purpose of these illustrative cases is to explore potential issues related to transparency, fairness, robustness, and accountability that could arise when deploying AI solutions in these contexts. By analysing these hypothetical scenarios, this study aims to provide insights into the proactive measures and strategies that manufacturing organisations can adopt to ensure AI trustworthiness.
6. Limitations and Bias Mitigation Strategies: As with any research, this study is subject to certain limitations. To address potential biases, several mitigation strategies were implemented throughout the research process. The possibility of selection bias in the illustrative case studies was reduced by ensuring a diverse representation of manufacturing sectors and AI application areas. To mitigate publication bias in the literature review, both peer-reviewed articles and grey literature sources (e.g., industry reports; white papers) were considered. The analytical subjectivity inherent in the toolkit evaluation was addressed through the use of a structured evaluation framework, clear evaluation criteria, and the involvement of multiple researchers in the analysis process to promote inter-rater reliability. While these strategies do not eliminate bias entirely, they significantly reduce its impact on this study’s findings.
7. Synthesis and Research Questions: Findings from the literature, toolkit evaluations, and case studies are synthesised to identify persistent gaps and emerging best practices. This study formulates open research questions to guide future inquiry, particularly regarding the operationalisation of trustworthy AI in dynamic, real-world manufacturing settings.

By combining a systematic review, critical analysis, and practical validation, this methodology aims to advance the understanding and implementation of trustworthy, ethical, and human-centric AI systems in manufacturing, supporting the broader objectives for manufacturing. The subsequent section examines the major technical, organisational, and ethical challenges that hinder AI adoption in Industry 5.0 manufacturing. It discusses issues such as black-box models, data quality, reliability, regulatory uncertainty, and workforce adaptation.

## 4. Challenges in AI Adoption for Industry 5.0

**Figure 6:**
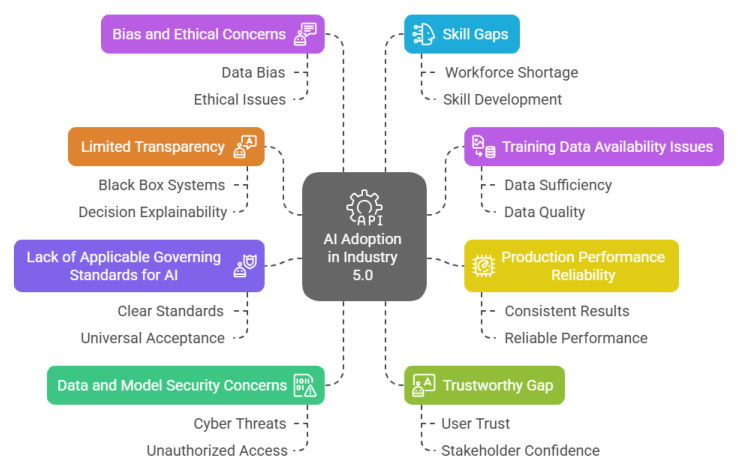
AI adoption challenges in Industry 5.0.

AI empowers the manufacturing industry to adapt to changing market demands, personalise products at scale, and strengthen supply chain resilience through advanced data analytics and automation. However, its successful integration into Industry 5.0 is not without challenges. As shown in Figure 6, various technical, organisational, and ethical barriers must be addressed to ensure AI’s seamless adoption and long-term impact [44].

1. Technical Challenges: A primary technical challenge is the “black box” nature of many AI models, which lack transparency and make it difficult for operators to trust or verify their decisions, raising concerns about accountability. The European Commission’s Ethics Guidelines stress the need for explainable and transparent AI to foster user trust [45,46]. Another issue is the shortage of high-quality, relevant data for training AI models. Poor or biased data can lead to inaccurate results and reinforce existing biases, limiting AI’s effectiveness in manufacturing [47]. Reliability is also a concern, as AI models that perform well in controlled settings may not replicate their success under real-world conditions due to variations in data distributions and unforeseen operational challenges, leading to inconsistent performance and affecting production quality and efficiency [48].
2. Security and Cybersecurity: Security concerns are paramount, as AI systems are susceptible to cyber threats that can compromise sensitive industrial data and disrupt operations. For example, adversarial attacks on machine learning models can manipulate outputs or cause system failures. The NIST Cybersecurity Framework and ISO/IEC 27001 provide standards for securing industrial AI systems, but their implementation in dynamic manufacturing environments remains challenging [49,50].
3. Ethical and Regulatory Challenges: Ethical considerations further complicate AI adoption. The potential for AI systems to perpetuate biases or make decisions that lack fairness necessitates the development of robust ethical frameworks. The European Commission’s guidelines advocate for AI that is lawful, ethical, and robust, ensuring adherence to principles such as fairness, accountability, and respect for privacy [45]. Regulatory uncertainty is a significant barrier, particularly where existing regulations conflict with AI optimisation. For instance, the General Data Protection Regulation (GDPR) mandates the right to explanation for automated decisions, which can conflict with the opacity of some machine learning models. This tension between data privacy and model transparency creates compliance challenges for manufacturers seeking to deploy advanced AI solutions [51]. The absence of standardised regulations and governing bodies for AI in manufacturing further exacerbates uncertainty, making it difficult for companies to ensure compliance and align with best practices. The European Commission’s ALTAI aims to provide actionable guidance to address these issues [52].
4. Organisational and Workforce Barriers: The human-centric approach of Industry 5.0 emphasises the importance of collaboration between AI systems and human workers. Bridging the skills gap through targeted education and training programs is vital to equip the workforce with the necessary competencies to effectively interact with AI technologies [53]. Organisational resistance to change, lack of digital maturity, and insufficient leadership support can also hinder successful AI adoption.
5. Barriers for SMEs versus Large Manufacturers: Small and medium-sized enterprises (SMEs) face unique barriers compared to large manufacturers. SMEs often lack the financial resources, technical expertise, and access to high-quality data required for effective AI implementation. The cost of acquiring, integrating, and maintaining AI systems can be prohibitive, and SMEs may struggle to attract or retain skilled personnel. In contrast, large manufacturers typically have greater capacity to invest in digital infrastructure, data management, and workforce development, enabling them to overcome many of these barriers more readily. As a result, the digital divide between SMEs and large enterprises may widen, limiting the broader impact of AI in the manufacturing sector [54].

## 5. Importance of AI Trustworthiness

AI systems are revolutionising various aspects of life, from recommending movies to diagnosing illnesses, assisting customers, and much more [55]. While AI offers a vast range of applications, its rapid advancement has also sparked significant concerns. The late Stephen Hawking once warned that “If not properly regulated, AI has the potential to become the greatest threat to humanity” [56].

Today, AI plays a crucial role in decision-making across multiple industries, but its outcomes are not always favourable. The growing reliance on AI brings a significant responsibility to ensure that these systems do not cause harm to humanity. However, there have been instances where AI has failed, leading to severe consequences. For example, the Correctional Offender Management Profiling for Alternative Sanctions (COMPAS) algorithm, widely used in the United States (US) to predict criminal recidivism risk, was found to exhibit racial bias against Black individuals [57]. A facial recognition system misclassified Black people due to poor-quality training data [58]. Similarly, a major tech company’s AI-driven resume screening system displayed bias against women [59]. These cases illustrate how bias can distort the decisions of black-box AI models, leading to unfair and harmful outcomes.

In some situations, AI has even resulted in physical harm due to system failures. One such case involved a self-driving car that struck and killed a pedestrian because its algorithm malfunctioned and failed to respond correctly when detecting a person on the road [60]. Moreover, the complexity of AI models makes it difficult to interpret their decision-making process, limiting their adoption and effectiveness. For instance, the research [61] found that despite their potential benefits, AI-powered medical diagnosis support systems have seen limited adoption among healthcare professionals. This reluctance stems from the lack of interpretability in these systems, reducing doctors’ trust and willingness to use them. AI systems have now reached a level of performance that allows them to be widely integrated into society. These technologies are already reshaping people’s daily lives [62]. However, despite their usefulness, this does not automatically mean they are reliable or trustworthy. A casual approach toward AI is unacceptable, especially in high-risk applications where a single wrong decision can have severe consequences. These systems can be fragile and prone to bias.

Marcus and Davis [63] provide a compelling example using facial recognition technology to illustrate the necessity of trustworthy AI. If such software is used for automatically tagging individuals in social media photos, a lower degree of accuracy may be tolerable. However, the same system becomes unacceptable when employed by law enforcement to identify suspects from surveillance images. This contrast highlights how AI is more readily adopted when errors do not pose serious risks to individuals or society.

To maximise the benefits of AI in critical applications and encourage broader adoption, it is essential to understand the reason behind the decision taken by an AI system. The following section outlines key requirements necessary to ensure AI systems are safe, reliable, and trustworthy.

## 6. Factors Defining AI Trustworthiness

In recent years, numerous research institutions, private companies, and government bodies have introduced various frameworks and guidelines aimed at ensuring that AI is trustworthy [61,64,65,66,67,68]. However, the overwhelming number of proposed principles has made it challenging to establish a unified set of standards. To address this issue, some researchers [15,69] have analysed and compared these principles to identify areas of consensus. Their findings indicate an emerging agreement on five key principles: transparency/explainability, justice and fairness, non-maleficence (which includes societal and environmental well-being), responsibility/accountability, and privacy. These principles appear more frequently in different frameworks compared to others.

To align with this analysis and adhere to one of the earliest government-backed AI frameworks, we have chosen the EU’s framework for trustworthy AI [61], which incorporates all five principles while also emphasising the human-centred aspect of AI.

The EU outlined three core guidelines that AI systems should follow to be considered trustworthy: they must be lawful, ethical, and robust. Lawfulness ensures that AI development, deployment, and usage comply with existing regulations. Ethical considerations require AI to respect human values and moral principles. Robustness emphasises that AI must be technically reliable while also adhering to legal and ethical standards. These guidelines provide a foundational structure for developing and deploying AI responsibly.

**Figure 7:**
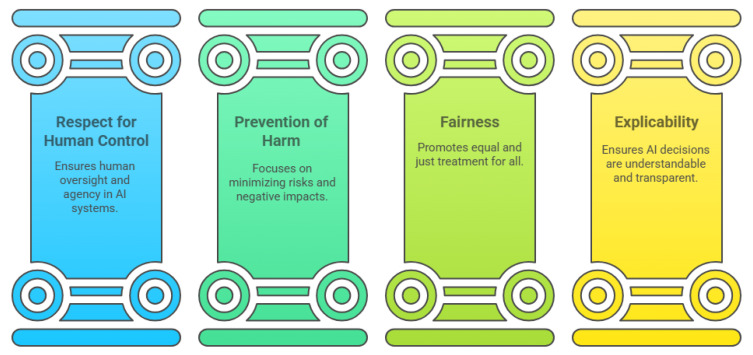
Trustworthy AI framework.

To operationalise these guidelines and enhance AI trustworthiness, the EU [61], introduced four ethical principles, each supported by seven key requirements, as summarised in Figure 7 [70,71]. The first principle, respect for human autonomy, ensures AI complements human decision-making rather than replacing it. The second principle, prevention of harm, guarantees that AI functions as intended without causing unintended damage to individuals or society. The third principle, fairness, ensures AI systems treat all individuals and social groups equitably, without bias or discrimination. Lastly, the fourth principle, explainability, ensures AI systems remain transparent and interpretable. These principles are explained through the following key requirements, which align with the aforementioned ethical principles:

1. Human Agency and Oversight: AI systems should support and enhance human decision-making rather than replace it. Human involvement should be proportional to the risks and societal impact of AI’s decisions [72,73].
2. Technical Robustness and Safety: AI systems must be reliable and function as intended. They should be capable of recovering from failures without harm and handle errors throughout the AI lifecycle. The system must also resist external threats and produce reproducible results [29].
3. Privacy and Data Governance: AI systems must safeguard user data throughout its lifecycle, ensuring compliance with data protection regulations like the General Data Protection Regulation (GDPR). Sensitive data must be protected from misuse [74].
4. Transparency: AI systems should be understandable, with decisions that can be explained, interpreted, and reproduced. Stakeholders should fully grasp the system’s performance and limitations [75,76].
5. Diversity, Non-discrimination, and Fairness: AI systems must ensure fairness, treating all societal groups equally and avoiding any form of discrimination, whether direct or indirect [77].
6. Societal and Environmental Well-being: AI systems should not harm society or the environment during their development, operation, or use [61].
7. Accountability: AI systems must be capable of justifying their decisions. There should be mechanisms for assigning responsibility for both correct and incorrect outcomes, along with regular audits to prevent harm [78].

### 6.1. Explainability

Explainability is key to ensuring that the rationale behind AI-driven decisions is clear, supporting transparency and making the system easier to interpret. This can lead to system improvements and stronger governance practices [79,80,81,82,83].

AI systems that offer clear explanations help identify flaws and vulnerabilities, contributing to the overall trustworthiness of the system [84,85]. Users have the right to understand how an AI system produces results, including insight into the system’s decision-making process, the data used to train it, and the criteria used to evaluate its outcomes [86,87,88]. Additionally, AI systems should offer explanations that cater to a wide range of users, each with varying levels of expertise and specific needs [89]. When users comprehend the reasons behind an AI system’s decisions, their trust in the system increases [90]. It is important to recognise that explanations vary depending on their intended purpose and the user’s background, resulting in different approaches to interpretability, such as global and local interpretability [91,92]. Global interpretability aims to explain the overall workings of an AI system, providing a high-level view of how decisions are made. This type of interpretability is typically used in large-scale applications like climate modeling [93], where practical challenges arise due to its scale. On the other hand, local interpretability is more focused on explaining individual decisions made by the AI system, offering more immediate and context-specific insights. The timing and relevance of these explanations are determined by the data used and the stage of decision-making [94,95]. Local interpretability can be further divided into two types: ex ante and ex post explanations [96]. Ex ante explanations describe how the system works and is designed before it is used, ensuring that it is adequately tested and reliable. Ex post explanations, however, clarify the reasons behind decisions after they are made, validating the assumptions established by the ex ante explanations [97]. As outlined by ISO [98], both ex ante and ex post explanations are critical components of an AI system’s trustworthiness through transparency and interpretability.

**Figure 8:**
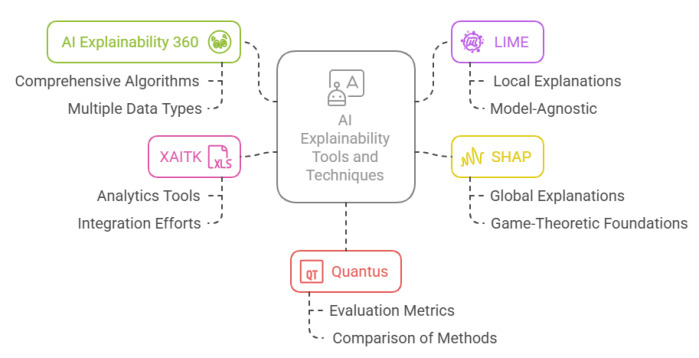
Explainability AI toolkits.

Current methods for ensuring explainability primarily address the needs of developers and designers, aiding in debugging and oversight [99]. However, more suitable approaches are needed to address the needs of non-expert users, bridging the gap between transparency and actual implementation [100]. In this context, the toolkits highlighted in Figure 8 and explained in Table 1 provide a variety of approaches to AI explainability, each designed to tackle different aspects of model transparency. For example, AI Explainability 360 (AIX360) and Local Interpretable Model-agnostic Explanations (LIMEs) focus on providing local explanations and enhancing trust through user-friendly models, which can be particularly helpful for non-expert users who may not have a deep understanding of machine learning. On the other hand, Shapley Additive Explanations (SHAPs) offers both global and local explanations that help users understand feature importance in a more comprehensive manner, which could be useful in settings requiring a higher degree of interpretability.

Furthermore, concerns about privacy and security can deter organisations from adopting AI solutions. Therefore, approaches that guarantee explainability while safeguarding privacy and security must be carefully developed [106,107,108,109]. Some of these toolkits, such as Quantus and Explainable AI Toolkit (XAITK), focus on evaluating and ensuring the robustness of explanation methods, which could be crucial for addressing privacy concerns by ensuring the fairness and transparency of AI systems without exposing sensitive data.

### 6.2. Accountability

To prevent algorithmic decision-making from leading to harmful outcomes, it is crucial to carefully oversee the design, deployment, and operation of these algorithms. Since algorithms are computer programs trained on data, those involved in their creation and user must take responsibility for any unintended consequences that arise [110,111,112]. In [78], the author characterises accountability as a collaborative effort, where different stakeholders are assigned responsibilities at various stages of the AI lifecycle. Essentially, ensuring accountability in algorithmic decision-making requires evaluating these systems against relevant standards and clearly defining the roles of those responsible for their development.

The increasing dependence on algorithmic decision-making (DM), particularly in high-risk environments, emphasises the need for strong accountability mechanisms. These algorithms must be designed, developed, and implemented in a reliable and secure manner to prevent potential failures. System malfunctions can have severe consequences, as demonstrated by the Boeing aircraft crash, which resulted in 346 fatalities due to software defects [113]. Similarly, Volkswagen encountered significant challenges with the software architecture of its electric vehicles, and a facial recognition system exhibited bias, disproportionately impacting women and individuals with darker skin tones [114]. Effective monitoring of these algorithms could help prevent such issues. However, assigning responsibility for these failures is complex—should the blame fall on developers, data collectors, or users trained to operate the system? The ambiguity surrounding accountability highlights the necessity for a well-structured framework [115,116].

Several strategies can enhance accountability in algorithmic DM. These include incorporating accountability measures into the algorithm’s design, increasing transparency, and enforcing stringent regulations and policies to improve oversight. Since accountability is a dynamic process [117,118], establishing it requires comprehensive governance throughout the AI lifecycle and active collaboration among all stakeholders [119]. However, pinpointing liability in the event of a system failure is challenging, as multiple parties are typically involved in the development process. Accountability measures should be tailored to specific applications, as a universal framework may not be suitable for all domains. To enhance governance, it is recommended to implement context-specific accountability strategies [98]. For example, ISO standards address accountability in medical AI systems and AI-driven hiring tools. In medical AI, healthcare providers bear responsibility for any harm caused, as they are experts in their field and the system is intended only to support their decision-making. Conversely, in AI-based recruitment systems, users are not held accountable for negative outcomes since they lack insight into why their application was rejected. This distinction underscores the importance of designing accountability measures that align with the specific context of each application [120].

### 6.3. Fairness

AI-driven systems and algorithms process vast amounts of data and logical rules to perform specific tasks and support decision-making. Given the significant role these systems play in everyday activities and operations, it is crucial to ensure they function without bias. A fair AI system should not discriminate against any individual or societal group [77]. The concept of fairness is closely aligned with ethical principles and moral values [121,122,123,124].

When AI systems are designed, developed, implemented, or monitored unfairly, they can produce harmful outcomes. Numerous cases illustrate the consequences of biased AI. For example, a judicial system was found to incorporate a flawed risk assessment tool that disproportionately discriminated against individuals with darker skin tones [125]. Similarly, a prominent technology company faced scrutiny for using a biased hiring algorithm that disadvantaged women [126]. Research has also revealed that certain predictive analytic tools used in child maltreatment screenings unfairly discriminated against marginalised groups based on race and socioeconomic status [127]. Several factors influence the trustworthiness of AI, including biases in data, models, and evaluation processes. Given the critical importance of fairness in AI, various studies have attempted to define the concept, yet there is no universally accepted definition. Some researchers have analysed and compared different interpretations of fairness in AI [122]. Generally, fairness in AI is context-dependent, meaning its definition varies based on how and where AI is applied. The two primary categories of fairness in AI are individual fairness and group fairness [122]. Individual fairness ensures that individuals within the same category receive consistent predictions [128]. This concept is associated with fairness through awareness [128] or unawareness [129], as well as counterfactual fairness [130,131]. On the other hand, group fairness focuses on equitable treatment across different societal groups [132]. Various methods are used to evaluate fairness in AI, such as demographic parity, which ensures balanced representation across groups, equalised odds, which accounts for fairness in prediction outcomes, equal opportunity, which focuses on equitable access to favourable results, and conditional statistical parity, which adjusts fairness based on specific conditions [131,133,134,135].

Beyond defining fairness, various approaches have been developed to promote fairness in AI. However, identifying a single universal method to detect and eliminate all types of bias remains challenging. In [136] the authors highlighted the need for further research to explore different perspectives on fairness, particularly within AI applications, as certain systems may be more vulnerable to specific biases than others. Establishing comprehensive frameworks and policies that define fairness in AI based on application context is essential. Additionally, stakeholders may have differing interpretations of fairness, emphasising the need for inclusive discussions to enhance AI trustworthiness. Strengthening testing protocols and implementing effective measures to detect and mitigate bias in AI systems is also vital [137].

**Figure 9:**
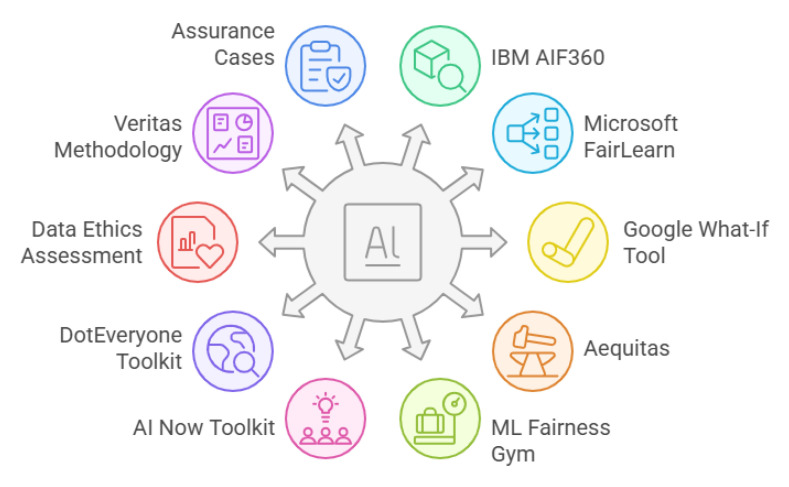
AI fairness toolkits.

To effectively address bias and ensure fairness in AI systems, various toolkits have been developed to assess and mitigate discriminatory outcomes. These toolkits provide diverse methodologies for evaluating fairness, offering both technical and ethical approaches. The Table 2 and Table 3 presents a comparative analysis of prominent AI fairness toolkits, highlighting their strengths, limitations, and application areas. Understanding these toolkits can be helpful for selecting the most suitable framework based on the specific needs of an AI system. Additionally, Figure 9 provides a visual representation of this comparison, further aiding in the evaluation and selection process.

### 6.4. Robustness

Robustness refers to the capability of an algorithm or system to handle execution errors, unexpected inputs, or unfamiliar data effectively. It is a crucial factor influencing the dependability of AI systems in practical settings. Insufficient robustness can lead to unintended consequences or hazardous behaviour, compromising both safety and trust. Within the domain of machine learning, robustness covers various aspects. In this review, we categorise AI system vulnerabilities into three primary levels: data, algorithms, and system robustness.

1. Data Level Robustness: A model trained on limited datasets that do not reflect real-world variations may suffer significant performance degradation. One major challenge is a distributional shift, where the data seen during deployment differs from the training data, affecting model reliability [147]. This issue is particularly concerning in safety-critical domains. For example, in autonomous driving, AI models must function under a range of environmental conditions. While a system trained in sunny weather may perform well, its effectiveness in night time or rainy conditions could be severely reduced. To address this, researchers and industry professionals employ extensive testing and development strategies to improve AI perception under varying weather conditions, ensuring consistent performance [148,149].
2. Algorithm-Level Robustness: AI models can be vulnerable to adversarial attacks, where maliciously modified inputs deceive the system. These attacks have raised concerns in both academia and industry, leading to extensive research on threat classification and defence mechanisms [150,151,152,153,154]. Adversarial attacks can be categorised based on their timing:
  - Decision-Time Attacks: These involve modifying input samples in real-time to manipulate the model’s predictions. Attackers may use such methods to bypass security mechanisms or impersonate legitimate users [155].
  - Training-Time Attacks (Poisoning Attacks): In this approach, adversaries introduce deceptive samples into the training data, influencing the model’s learning process and altering its behaviour in specific situations [155].
  Another important classification is based on the space in which attacks are conducted:
  - Feature-Space Attacks: Traditional adversarial methods directly alter input features to deceive the model.
  - Problem-Space Attacks (Entity-Based Attacks): Instead of modifying digital data, attackers alter physical objects to manipulate AI recognition. For example, a person wearing specially designed adversarial glasses could bypass a facial recognition system [156,157]. Apart from adversarial attacks, model stealing (exploratory attacks) is another significant threat. These attacks do not directly alter model behaviour but extract knowledge about the AI system, which can later be exploited to craft more effective adversarial samples [158].
3. System-Level Robustness: AI systems must be designed to handle a wide range of unexpected or illegal inputs in real-world applications. Practical cases include the following:
  - Unanticipated Inputs: For instance, an image with an extremely high resolution might cause an AI-based image recognition system to crash.
  - Sensor Interference: In autonomous vehicles, a lidar system might misinterpret signals from other vehicles, leading to corrupted input data.
  - Spoofing Attacks: Attackers may use fake inputs—such as printed photos or masks—to deceive biometric authentication systems, raising security concerns [159]. To mitigate these risks, defensive mechanisms are categorised as either proactive or reactive [160]. Proactive defences aim to strengthen AI models against diverse inputs, making them inherently robust. Reactive defences focus on detecting adversarial samples or identifying anomalies in data distribution.
4. Evaluating Robustness: Assessing robustness is crucial for detecting vulnerabilities and managing risks. Two primary evaluation methods are robustness testing and mathematical verification.
  - Robustness Testing Testing plays a key role in validating AI robustness, just as it does in traditional software development. Techniques such as monkey testing—which uses randomised inputs to check system stability—can be applied to AI models [161]. Additionally, software testing methodologies have been adapted to assess AI resilience against adversarial attacks [162,163].
    Another common method is performance testing (benchmarking), which evaluates model robustness using test datasets with varying distributions. One widely used metric is the minimal adversarial perturbation, which measures the smallest modification needed to mislead an AI model. Another key evaluation metric is the attack success rate, which reflects how easily an adversary can compromise the system [164,165].
  - Mathematical Verification Borrowed from formal verification methods, mathematical validation techniques are increasingly used to assess AI robustness. For instance, researchers derive certified lower bounds on the minimum distortion required for an adversarial attack—a measure of how resistant a model is to adversarial manipulations [166,167].

**Figure 10:**
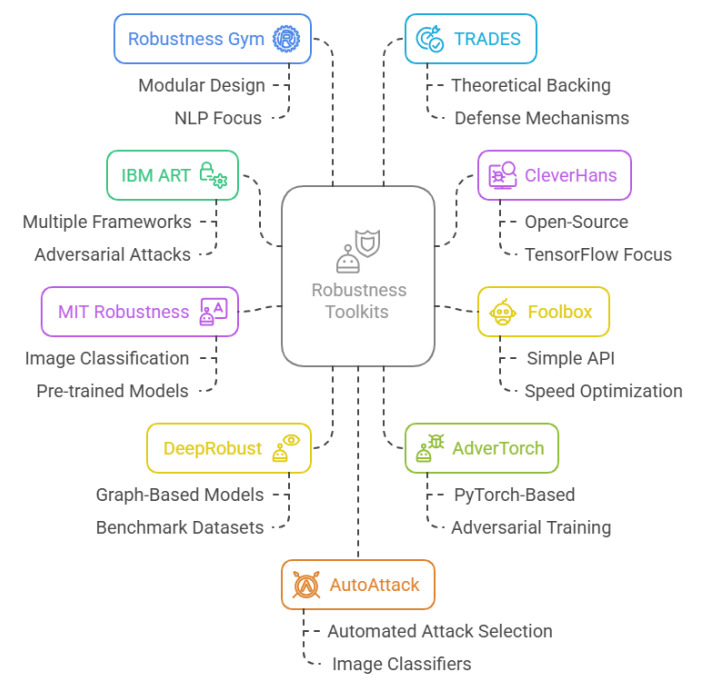
AI robustness toolkits.

To enhance AI robustness, researchers and practitioners have developed specialised toolkits that help assess, mitigate, and defend against various vulnerabilities. These toolkits provide methods for robustness testing, adversarial attack detection, and model hardening, ensuring AI systems perform reliably across different conditions. Table 4 presents a comparative overview of key robustness toolkits, highlighting their functionalities, advantages, limitations, and practical use cases. Figure 10 further illustrates this comparison through a visual representation, providing additional clarity for evaluating these toolkits. The next section analyses how these trustworthiness factors are addressed across the entire AI pipeline, from data collection to model deployment. It formulates research questions and highlights practical challenges and best practices for ensuring trustworthy AI in real-world manufacturing settings.

## 7. Challenges in the AI Pipeline: From Data Collection to Model Deployment

While the principles of AI trustworthiness—transparency, fairness, robustness, and accountability—are well established in the literature, their practical realisation in manufacturing environments presents unique challenges and opportunities. This review bridges the theoretical and practical dimensions by mapping these core principles onto concrete factory-level scenarios. For example, transparency is operationalised through the deployment of explainable AI (XAI) tools that allow production engineers to interpret and validate machine learning predictions for quality control, thereby increasing trust in automated inspection systems. Fairness is addressed by monitoring and mitigating biases in predictive maintenance algorithms, ensuring that all equipment types and production lines receive equitable attention, rather than favouring those with more historical data. Robustness is exemplified by the implementation of adversarial testing protocols in digital twins, which simulate unexpected disruptions—such as sensor failures or supply chain shocks—to assess the resilience of AI-driven decision systems. Accountability is reinforced through the establishment of clear audit trails and responsibility matrices, enabling traceability of AI-driven decisions and facilitating compliance with regulatory standards. The practical realisation of these strategies often necessitates interdisciplinary collaboration. For instance, in the deployment of predictive maintenance systems, data scientists, manufacturing engineers, and ethicists have worked together to design algorithms that are not only technically robust but also transparent and fair in their recommendations. Such collaborations ensure that AI solutions are informed by domain expertise, ethical considerations, and operational realities, thereby enhancing both societal impact and user acceptance. By providing these scenario-based insights, this review demonstrates how the abstract dimensions of AI trustworthiness can be translated into actionable strategies and best practices for factory operations. This theoretical–practical integration not only supports the adoption of trustworthy AI in manufacturing but also aligns with the broader objectives of Industry 5.0, which emphasise human-centricity, sustainability, and ethical responsibility in industrial innovation. However, even with these strategies in place, manufacturing is undergoing a radical transformation driven by AI, particularly within the framework of Industry 5.0, which emphasises human–machine collaboration. AI technologies are central to this transformation, enabling smarter, more efficient, and adaptable operations [1]. Yet, the adoption of AI is not without challenges. Each step in the AI pipeline—from data collection to model deployment and post-deployment monitoring—presents unique hurdles that impact the overall trustworthiness and effectiveness of AI solutions. Addressing these challenges requires a careful balance between technical innovation, robust ethical frameworks, and organisational transformation [178].

**Figure 11:**
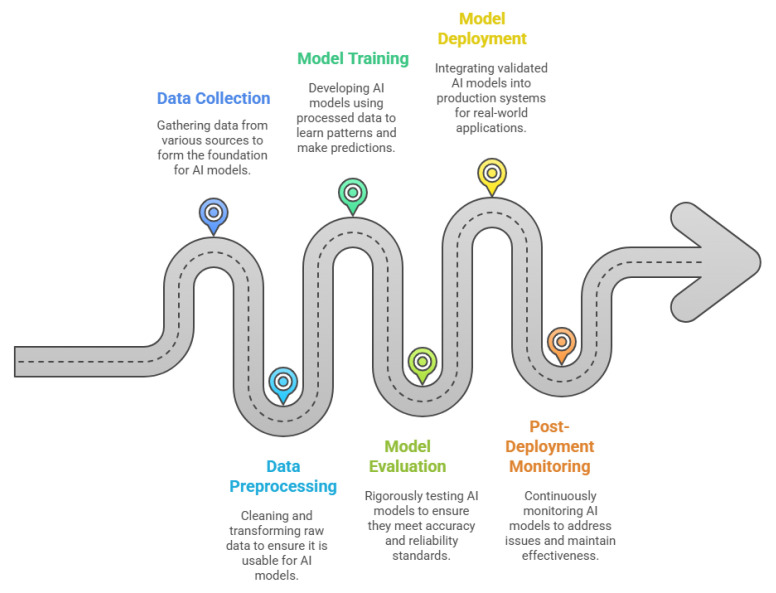
AI model development stages.

The development of AI models follows a structured pipeline shown in Figure 11, ensuring a systematic approach to creating reliable and trustworthy systems. The process begins with data collection, where data is gathered from various sources such as servers and IoT devices. This data forms the foundation for building AI models. Once collected, the data is securely stored in data storage systems, including databases like MySQL, PostgreSQL, and MongoDB, as well as cloud-based storage buckets such as Amazon S3, Google Cloud Storage, and Microsoft Azure Blob Storage. These systems ensure the data is organised, accessible, and ready for further processing [179].

In the data processing stage, the raw data is filtered to remove noise and irrelevant information, and it is transformed into a usable format. This step ensures the data is clean, consistent, and ready for analysis. The processed data is then stored again in storage systems to maintain its integrity and accessibility for subsequent stages [180].

The next step is model training, where analysts and machine learning engineers use the processed data to train AI models. This involves designing algorithms that learn patterns and make predictions based on the data. For example, in manufacturing, AI models may be trained to predict equipment failures, optimise production schedules, or improve quality control. After training, the models undergo model evaluation, where their performance is rigorously tested to ensure they meet the required standards of accuracy, fairness, and robustness. This step is critical in manufacturing, as any inaccuracies in predictions or decisions can lead to costly disruptions or defects [181].

Finally, the validated models are integrated into production systems during the model deployment stage. Here, the models are used to perform real-world tasks, such as predictive maintenance, quality inspection, or supply chain optimisation. Post-deployment monitoring ensures that the models continue to perform effectively, addressing issues such as data drift, performance degradation, and cybersecurity risks. For instance, in manufacturing, continuous monitoring can help detect changes in production conditions or equipment behaviour that may affect the model’s accuracy [182].

This structured pipeline ensures a systematic approach to AI development, enabling the creation of transparent and reliable AI systems in manufacturing. However, each stage of this pipeline presents unique challenges, such as ensuring data quality, addressing biases, and maintaining model robustness in dynamic manufacturing environments. These challenges must be addressed to fully realise the potential of AI in manufacturing and to build systems that are not only effective but also trustworthy and aligned with the principles of Industry 5.0. As AI systems progress through each stage of the manufacturing pipeline, trustworthiness can gradually erode—a process known as “trust leakage.” Small issues like data bias or reduced robustness, if not addressed early, may be amplified in later stages [27]. In the following subsections, we discuss how trust leakage can arise at each phase and strategies to mitigate these risks.

### 7.1. Data Collection

The data collection stage is fundamental to manufacturing AI, with data sourced from IoT sensors, legacy equipment, and digital systems. However, manufacturing data is often noisy, incomplete, and inconsistent, making it especially vulnerable to bias and privacy issues. If these challenges are not addressed early, they can propagate through the pipeline and compromise the fairness and reliability of AI models [183]. See Box 1.

Box 1. **Illustrative example 1.**

**Figure 12:**
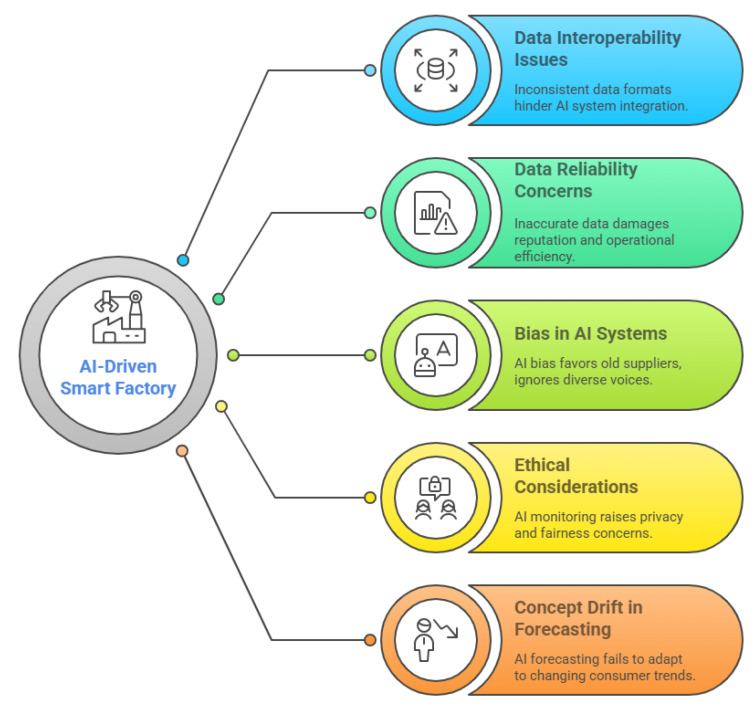
Navigating AI challenges in smart manufacturing.

A global leader in sustainable manufacturing shown in Figure 12 has implemented an AI-driven smart factory to produce environmentally friendly products. The factory integrates AI across its operations, including supply chain management, production optimisation, predictive maintenance, and employee monitoring, with the goal of improving efficiency, reducing costs, and meeting sustainability targets. However, as the factory scales its AI systems, several challenges emerge. The organisation collects data from multiple sources, such as supplier databases, IoT sensors, and customer feedback, but faces issues with data interoperability due to inconsistent formats and schemas. For example, supplier data may use different terminologies, and IoT sensors from various vendors often produce incompatible data, leading to errors in supplier evaluation and production scheduling. Additionally, the reliability of external data sources becomes a concern when inaccurate information, such as incorrect carbon footprint data for materials, damages the organisation’s reputation when the error is discovered. Bias in historical data further complicates matters, as supplier selection models may favour long-term suppliers over new, innovative ones, and shopfloor automation systems might fail to recognise diverse accents and voices due to insufficiently inclusive training data. Predictive maintenance systems can also exhibit regional bias, relying on data from older machines predominantly used in one region, which results in inaccurate predictions for newer machines. Moreover, noisy or incomplete data from IoT sensors can cause false alarms in maintenance systems, leading to unnecessary production delays. Ethical concerns arise as the AI system monitors employee movements to ensure safety compliance but also flags workers for “low performance” based on arbitrary metrics, disproportionately affecting certain groups and raising privacy concerns. Finally, the AI system for demand forecasting struggles with concept drift, failing to adapt to sudden shifts in consumer preferences, which results in stockouts and lost sales.

The above example highlights the challenges, such as data bias, interoperability, and privacy, that can arise during the data collection steps. Therefore, this research raises the following questions related to this step:

- RQ1: How can data producers and owners implement interoperable data schemas to ensure data integrity?
- RQ2: What mechanisms best facilitate the extraction of unbiased, informative datasets from complex environments?
- RQ3: What types of biases are present in manufacturing datasets and data collection processes, and what strategies can be used to detect and address them while maintaining optimal performance?
- RQ4: What methods can be employed to gather unbiased and informative datasets from shop floor environments where human involvement is significant?
- RQ5: How can workers with limited AI expertise effectively evaluate algorithms for bias and fairness?

### 7.2. Data Preprocessing

Data augmentation and preprocessing are essential for preparing manufacturing data—often sourced from IoT sensors, machine logs, and manual entries—for AI models. These steps clean, balance, and transform raw data to ensure reliability and accuracy. However, aggressive cleaning or augmentation can unintentionally introduce or amplify bias, impacting the fairness and robustness of downstream models [184]. See Box 2.

Box 2. **Illustrative example 2.**

**Figure 13:**
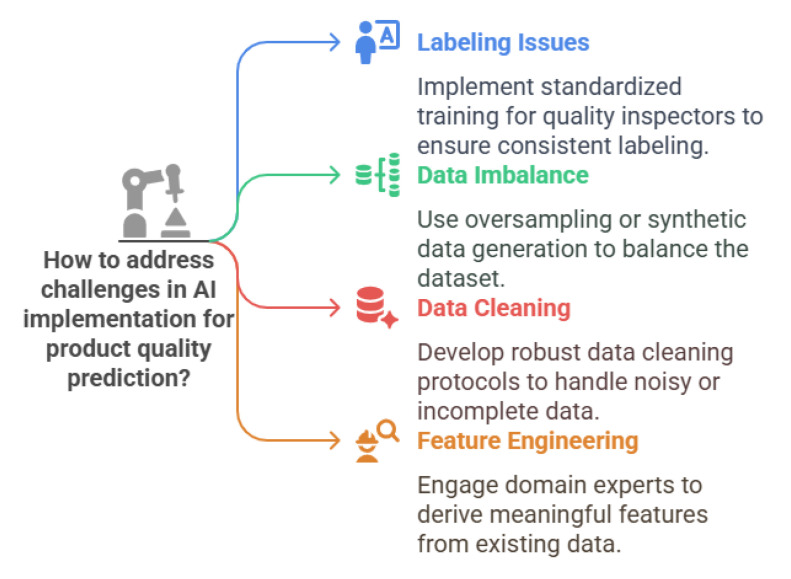
AI challenges in manufacturing.

Imagine a manufacturing company that produces automotive parts and wants to implement an AI system to predict product quality based on production parameters. The company collects data from various sources, including IoT sensors on machines, manual quality checks, and supplier records. However, several challenges arise shown in Figure 13 during data augmentation and preprocessing:

- Labelling Issues: The company needs labelled data to train its AI model, but quality labels are subjective and depend on the expertise of the quality inspectors. For instance, one inspector might classify a product as “acceptable”, while another might label it as “defective” under similar conditions. This inconsistency leads to uncertain labels, which can affect the model’s accuracy.
- Data Imbalance: Most of the products meet quality standards, resulting in an imbalanced dataset where defective products are under-represented. This imbalance can cause the AI model to overlook rare but critical defects.
- Data Cleaning: The IoT sensors occasionally produce noisy or incomplete data due to hardware malfunctions or network issues. For example, temperature readings from a sensor might show sudden spikes that do not reflect actual conditions, leading to incorrect predictions.
- Feature Engineering: The production team hypothesises that the humidity level in the factory might influence product quality. However, this data is not directly available and needs to be derived from existing environmental data, requiring domain expertise to create meaningful features.

To address the identified challenges in data preprocessing and augmentation for AI applications in manufacturing, the following research questions are proposed:

- RQ6: What methodologies can be developed to ensure consistent and accurate labelling of manufacturing data, particularly in scenarios where labels are subjective or context-dependent?
- RQ7: What are the most effective practices for addressing imbalanced datasets in manufacturing, and how can synthetic data generation techniques be optimised for such applications?
- RQ8: How can automated approaches be designed to detect and rectify noisy or incomplete data originating from IoT sensors and other manufacturing data sources?
- RQ9: What strategies can be employed to integrate domain knowledge into feature engineering processes while minimising the risk of introducing additional biases?
- RQ10: What frameworks or methodologies can be developed to identify and mitigate biases in manufacturing datasets, ensuring fair and unbiased AI-driven predictions?

### 7.3. Developing AI Models

Model development in manufacturing involves model selection, training, and deployment—each with unique challenges for trustworthiness. This stage is especially vulnerable to overfitting, lack of transparency, and bias amplification, particularly when performance is prioritised over interpretability. Addressing these risks is essential to ensure reliable and effective AI models.

1. Model selection is a crucial phase where the type of machine learning model is chosen, as it directly influences the model’s interpretability and performance. Simpler models, such as decision trees, are often preferred in manufacturing due to their ease of understanding and transparency. However, this preference can sometimes lead to reduced accuracy, creating a trade-off between interpretability and performance. Additionally, computational limitations, particularly in resource-constrained environments, can restrict the use of more advanced models, further complicating the selection process [181].
2. Model training is another critical phase, where hyperparameters such as the depth of decision trees or the number of layers in a neural network are fine-tuned to enable the model to learn patterns effectively from the data. This phase is resource-intensive, with high computational and environmental costs, especially for large-scale models. The process also requires skilled professionals to manage training effectively and avoid suboptimal results. While automated machine learning (AutoML) tools can simplify the training process, they often introduce challenges such as reduced transparency, potential bias, and overfitting, which can compromise the trustworthiness of the model [185].
3. Deployment phase focuses on integrating the model into real-world operations and ensuring its continued relevance. A significant challenge in this phase is addressing concept drift, where changes in the data distribution over time can render the model’s predictions inaccurate. To mitigate this, organisations must implement mechanisms to detect when updates are needed and determine whether incremental updates or full retraining is more appropriate. This phase requires careful monitoring and adaptation to ensure the model remains effective in dynamic manufacturing environments [186]. See Box 3.

Box 3. **Illustrative example 3.**

**Figure 14:**
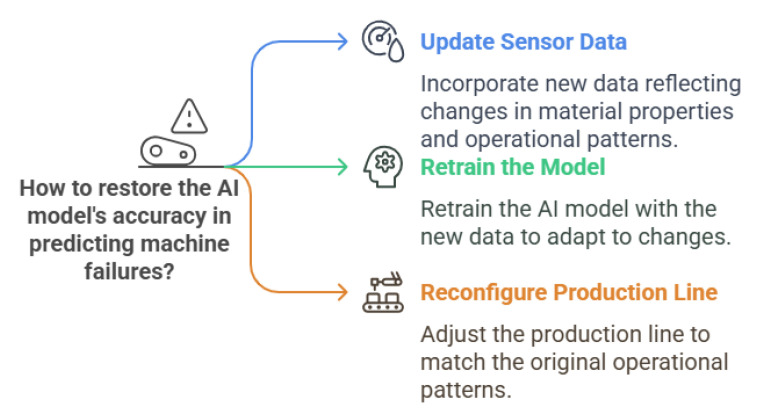
AI challenges in manufacturing.

A large automotive manufacturing company developed an AI model to predict machine failures on its assembly line, aiming to reduce unplanned downtime and optimise maintenance schedules. Initially, the model performed exceptionally well, using data from sensors monitoring machine vibrations, temperature, and operational cycles. The predictions allowed the company to schedule maintenance proactively, significantly reducing production delays. However, after several months, the model’s accuracy began to decline, leading to unexpected machine breakdowns. Upon investigation, the team discovered that the supplier of a critical machine component had changed, resulting in slight variations in material properties. These changes in sensor readings were outside the scope of the model’s training. Additionally, the production line was reconfigured to accommodate a new vehicle model, which altered the operational patterns of the machines, as shown in Figure 14.

To address the challenges highlighted above, this paper raises several important research questions.

- RQ11: How can manufacturing practitioners balance the trade-off between model interpretability and performance?
- RQ12: What are the most effective methods for detecting and managing concept drift in industrial applications?
- RQ13: How can organisations account for the environmental and financial costs of training AI models?

## 8. Conclusions and Future Directions

AI is fundamentally transforming the manufacturing sector by enabling smarter, more efficient, and adaptive processes. As manufacturers increasingly depend on AI for decision-making, the importance of trustworthiness in these systems becomes paramount. Trustworthy AI is not only about technical accuracy but also about ensuring transparency, fairness, robustness, and accountability. These principles are essential to prevent unintended consequences, such as biased decisions, lack of explainability, or system failures that could disrupt operations and erode stakeholder trust.

This paper has provided a comprehensive review of the current landscape of AI trustworthiness in manufacturing. It has highlighted the progress made in developing frameworks and tools for explainability, bias mitigation, and robust model deployment. However, it is clear that manufacturing environments are highly dynamic and complex, and no single solution can address all challenges. The effective implementation of trustworthy AI requires ongoing attention to ethical considerations, human values, and close collaboration between engineers, domain experts, and end-users. Only through such a holistic approach can the manufacturing industry fully harness the benefits of AI while minimising risks and ensuring responsible innovation.

Looking forward, the journey toward trustworthy AI in manufacturing must continue to evolve. Continuous monitoring and adaptation of AI models will be necessary to maintain their accuracy and fairness as manufacturing data and environments change over time. There is also a growing need to develop and adopt frameworks that integrate ethical, environmental, and financial considerations into every stage of the AI lifecycle. This integration will help ensure that AI systems not only perform well but also align with broader societal and sustainability goals.

Interdisciplinary collaboration will play a crucial role in this evolution. By bringing together AI specialists, manufacturing professionals, and ethicists, the industry can develop solutions that are both technically robust and ethically sound. Furthermore, regulatory compliance with emerging standards, such as the EU AI Act and ISO guidelines, will be essential for responsible AI deployment and for building stakeholder confidence.

A significant next phase for this research will involve the practical testing and evaluation of leading AI trustworthiness toolkits within real manufacturing scenarios. By rigorously assessing these toolkits, this research aims to identify which solutions are most effective and user-friendly in practice. The insights gained from this phase will provide actionable recommendations for industry adoption and will help bridge the gap between theoretical frameworks and real-world application.

While this study provides a comprehensive overview of AI trustworthiness in manufacturing, several limitations should be acknowledged. Methodologically, the reliance on literature review and illustrative, hypothetical case studies may limit the generalisability of the findings, as real-world complexities and sector-specific nuances may not be fully captured. Conceptually, the operationalisation of AI trustworthiness, though grounded in established frameworks, may be subject to interpretation and may not encompass all emerging dimensions as the field evolves. Contextually, the focus on manufacturing means that insights may not directly translate to other sectors or geographic regions with different regulatory, cultural, or operational landscapes. These limitations highlight the need for further empirical validation and cross-sectoral analysis.

To advance the field, future research should focus on developing robust, quantitative methods for assessing and benchmarking AI trustworthiness in manufacturing environments. This includes the creation of standardised metrics and tools for trust quantification, enabling objective comparison across different AI systems and deployment contexts. Comparative studies of AI regulatory frameworks across sectors and regions are also needed to identify best practices and inform the development of harmonised, context-sensitive standards. Additionally, longitudinal research tracking the long-term impacts of AI adoption on workforce dynamics, ethical outcomes, and organisational performance will provide valuable insights for both policymakers and industry leaders. Finally, there is a need for in-depth, real-world pilot studies that evaluate the effectiveness of trustworthiness toolkits and frameworks in diverse manufacturing settings, thereby closing the gap between theoretical advances and practical implementation.

## Figures and Tables

**Table 1:** Comparison of AI explainability toolkits.

Toolkit	Pros	Cons	Use Cases
AI Explainability 360 (AIX360) [101]	Comprehensive set of algorithms covering various explanation dimensions.	Steep learning curve due to broad feature set.	Understanding and interpreting predictions from complex machine learning models.
	Supports multiple data types, enhancing versatility.	Some algorithms may require substantial computational resources.	Ensuring transparency and trustworthiness in AI-driven decision-making processes.
	Developed by IBM, ensuring reliability and community support.		
LIME (Local Interpretable Model-agnostic Explanations) [102]	Provides local explanations by approximating complex models with simpler ones.	Local explanations may not fully capture global model behaviour.	Explaining individual predictions in domains like healthcare and finance.
	Model-agnostic; applicable to various machine learning models.	Performance can be affected by noisy data, leading to inconsistent interpretations.	Assisting in debugging and improving model performance by understanding specific decision paths.
	Enhances user trust through understandable explanations.		
SHAP (Shapley Additive Explanations) [103]	Offers both global and local explanations, providing a comprehensive view of model behaviour.	Computationally intensive, especially with large datasets and complex models.	Assessing feature importance in predictive models.
	Based on solid game-theoretic foundations, ensuring consistent and fair feature importance values.	Requires careful handling of feature interactions to avoid misleading interpretations.	Enhancing model transparency in sectors like finance and healthcare by elucidating the impact of individual features on predictions.
XAITK (Explainable AI Toolkit) [104]	Provides a suite of tools for analysing and understanding complex machine learning models.	May require integration efforts with existing workflows.	Analysing and interpreting complex machine learning models across various domains.
	Includes analytics tools and methods for interpreting models, supporting various explanation techniques.	Documentation and community support might be less extensive compared to more established toolkits.	Supporting research and development in AI transparency and accountability.
Quantus [105]	Offers a collection of evaluation metrics for assessing the quality of explanations.	Primarily focused on evaluating explanations rather than generating them.	Evaluating the effectiveness of different explainability methods.
	Facilitates the comparison of different explanation methods.	May require additional tools or methods for generating explanations.	Assisting researchers in selecting appropriate explanation techniques for their models.
	Aids in identifying the most effective explanation techniques for specific models.		

**Table 2:** Summary of AI fairness toolkits (Part 1). These toolkits are used to assess and mitigate bias in AI systems, ensuring fairness in decision-making processes.

Toolkit Name	Advantages	Disadvantages	Use Cases
IBM AI Fairness 360 (AIF360) [138]	Open-source toolkit for bias detection and mitigation.	Focuses primarily on fairness, may not address other ethical AI dimensions.	Evaluating and mitigating bias in hiring, loan approvals, and other decision-making systems.
	Provides a comprehensive set of metrics and algorithms for bias mitigation.	Can be complex to implement in production systems.	Improving fairness in public services, education, and financial systems.
	Highly customisable and suitable for large-scale applications.	May require significant computational resources.	Ensuring fairness in automated systems such as hiring and loan approvals.
Microsoft FairLearn [139]	Provides fairness assessment and bias mitigation tools.	Requires technical expertise for implementation.	Fairness assessment in machine learning models, especially in enterprise settings.
	Supports multiple fairness metrics and mitigation strategies.	Limited documentation for non-technical users.	Ensuring fairness in predictive models used in hiring, finance, and healthcare.
	Enables easy integration with scikit-learn models.	Limited flexibility in non-enterprise applications.	Used for bias mitigation in sensitive decision-making systems.
Google What-If Tool [140]	Interactive tool for exploring model predictions and fairness.	Limited to TensorFlow models.	Analyzing fairness in predictive models, such as fraud detection and medical diagnoses.
	Allows visual exploration of bias and fairness across model predictions.	Not suitable for non-TensorFlow based models.	Used for exploring fairness in machine learning models for fraud detection and healthcare applications.
	Easy-to-use interface for non-technical stakeholders.	Can be time-consuming for large datasets.	Assessing fairness in machine learning applications for public policy and social justice.
Aequitas [141]	Focuses on bias and fairness in decision-making systems.	Limited scope for other ethical AI principles.	Bias detection in public policy and social justice applications.
	Designed to be used with real-world decision-making data.	Does not provide technical tools for bias mitigation.	Used for fairness assessments in hiring, criminal justice, and education systems.
	Strong documentation and user support.	Not highly customisable for complex AI systems.	Ensuring fairness in decision-making systems related to government and social issues.
ML Fairness Gym [142]	Simulates long-term impacts of fairness interventions.	Requires expertise in simulation modeling.	Evaluating fairness in dynamic systems like credit scoring and hiring.
	Provides an interactive environment for testing fairness interventions.	High computational cost for large simulations.	Used for understanding fairness in long-term decision-making systems.
	Supports a range of fairness interventions for experimentation.	Simulation results may not generalise to all real-world scenarios.	Assessing fairness in evolving applications such as credit scoring and insurance.

**Table 3:** Summary of AI fairness toolkits (Part 2). These toolkits are used to assess and mitigate bias in AI systems, ensuring fairness in decision-making processes.

Toolkit Name	Advantages	Disadvantages	Use Cases
AINow Algorithmic Impact Assessment Toolkit. [143]	Engages stakeholders in assessing fairness and ethical implications.	Limited technical tools for bias mitigation.	Assessing fairness in community-focused AI applications.
	Provides a structured framework for ethical AI assessments.	Limited to ethical assessments rather than technical solutions.	Used for impact assessments in AI systems affecting marginalised communities.
	Emphasises the importance of human oversight in AI decision-making.	Lacks deep technical fairness metrics and algorithms.	Assisting in responsible AI implementation in public services.
DotEveryone Consequence Scanning Toolkit	Open-source; minimal resources required; focuses on societal impacts.	Requires a strong facilitator, which may be a barrier for SMEs.	Conceptualising AI systems with societal and environmental considerations.
	Helps in identifying the societal consequences of AI deployments.	Primarily focused on societal impact rather than technical fairness.	Used for ethical evaluations of AI systems in public policy, education, and healthcare.
	Allows for early detection of ethical and social risks in AI systems.	Lacks comprehensive tools for technical fairness evaluation.	Ensuring societal considerations are addressed in AI-based systems.
Data Ethics Impact Assessment [144]	Integrates data ethics into AI development processes.	Limited to ethical assessments, not technical bias mitigation.	Ethical assessments in AI systems for public and private sectors.
	Focuses on the ethical implications of data usage and AI models.	May not address technical fairness challenges directly.	Used in ensuring data usage complies with ethical standards in sectors like healthcare and government.
	Provides an important framework for responsible AI development.	Does not provide tools for bias correction or mitigation.	Ensuring ethical and fair data usage in AI systems for social justice.
Veritas Fairness Assessment Methodology. [145]	Developed for financial systems; focuses on fairness and transparency.	Limited adoption outside the finance industry.	Fairness assessments in credit scoring and insurance systems.
	Strong focus on transparency and accountability in financial applications.	Not widely applicable to non-financial systems.	Used in fairness evaluations of automated financial decision-making systems.
	Tailored for regulatory and compliance environments.	Limited support for non-financial use cases.	Ensuring fairness in automated decision-making systems in banking and finance.
Assurance Cases for Fairness. [146]	Provides structured arguments for fairness claims.	Requires domain expertise and collaboration for effective implementation.	Ensuring fairness in AI systems for healthcare and education.
	Useful in establishing transparency and accountability for fairness claims.	May require significant resources to build effective assurance cases.	Used in verifying fairness in AI-based healthcare and educational systems.
	Focuses on providing evidence-based assurance for fairness.	Limited scalability to large AI systems.	Ensuring trust and accountability in AI systems in sectors with high public scrutiny.

**Table 4:** Comparison of AI robustness toolkits.

Toolkit	Pros	Cons	Use Cases
IBM ART [168]	Supports multiple ML frameworks	Can be complex to set up	Security testing for AI in finance & healthcare
	Offers both attacks & defences	Some attacks are slow	Adversarial training for robust AI models
	Includes explainability tools		
CleverHans [169]	Well-documented	Limited defence techniques	Evaluating deep learning model security
	Strong focus on adversarial attacks	Primarily TensorFlow-focused	Research on adversarial robustness
	Open-source and widely used		
Foolbox [170]	Simple API for adversarial attacks	Lacks built-in defence mechanisms	Red teaming for AI security
	Works with PyTorch, TensorFlow, JAX	Not as actively maintained as ART	Benchmarking model vulnerability
	Optimised for speed		
MIT Robustness [171]	Designed for adversarial training	Focused on image classification tasks	Adversarial training in computer vision
	PyTorch support	Limited support for non-vision models	Research in robustness techniques
	Provides pre-trained robust models		
DeepRobust [172]	Supports both graph and image-based AI models	Requires deep understanding of adversarial learning	AI security in social networks (graph AI)
	Covers attacks and defences	Not as widely adopted as ART or CleverHans	Robustness evaluation for medical AI models
	Provides benchmark datasets		
AdverTorch [173]	PyTorch-based attack toolkit	Native PyTorch support	Developing adversarial defences in PyTorch
	Various attack implementations	Limited to PyTorch ecosystem	Generating adversarial examples
	Focus on adversarial training		Robust training pipeline integration
Robustness Gym [174]	Robustness benchmarking suite	Modular and extensible	Evaluating NLP model robustness
	Supports data transformations	Primarily NLP-focused	Stress-testing AI models in production
	Model evaluation techniques		Enhancing general AI model reliability
TRADES [175]	Trade-off between robustness and accuracy	Strong theoretical backing	Improving robustness in DNNs
	Adversarial training framework	Requires deep ML expertise	Research on robust AI training methods
	Defence against adversarial perturbations		Defending against adversarial attacks
AutoAttack [176]	Ensemble adversarial attack method	Strong attack performance	Benchmarking adversarial robustness
	Automates attack selection	Limited flexibility for defences	Validating adversarial defences
	Works on image classifiers		Testing robustness in computer vision
RobustBench [177]	Maintains a leaderboard of robust models	Limited set of attacks compared to ART	Benchmarking AI robustness in academia
	Easy benchmarking of defences	Mostly vision-focused	Comparing adversarial defences
	Open-source		

## Data Availability

Not applicable.

## Abbreviations

The following abbreviations are used in this manuscript:

AI	Artificial Intelligence
GDPR	General Data Protection Regulation
AIX360	AI Explainability 360
SHAP	SHapley Additive exPlanations
LIME	Local Interpretable Model-agnostic Explanations
AIF360	AI Fairness 360
IBM ART	IBM Adversarial Robustness Toolbox
NIST	National Institute of Standards and Technology
OECD	Organisation for Economic Co-operation and Development
ISO	International Organization for Standardization
IEC	International Electrotechnical Commission
IEEE	Institute of Electrical and Electronics Engineers
ALTAI	Assessment List for Trustworthy Artificial Intelligence
EU	European Union
IoT	Internet of Things
ML	Machine Learning
XAITK	Explainable AI Toolkit
HVMC	High-Value Manufacturing Catapult
COMPAS	Correctional Offender Management Profiling for Alternative Sanctions
AutoML	Automated Machine Learning
NLP	Natural Language Processing
DNN	Deep Neural Network
CNN	Convolutional Neural Network
RMF	Risk Management Framework

## References

[1] Lu Y. (2017). Industry 4.0: A survey on technologies, applications and open research issues. J. Ind. Inf. Integr..

[2] Xu L.D., Duan L. (2019). Big data for cyber physical systems in industry 4.0: A survey. Enterp. Inf. Syst..

[3] Olsen T.L., Tomlin B. (2020). Industry 4.0: Opportunities and challenges for operations management. Manuf. Serv. Oper. Manag..

[4] Russell S.J., Norvig P. (2016). Artificial Intelligence: A Modern Approach.

[5] Mitchell T.M. (1997). Machine Learning.

[6] Lee J., Bagheri B., Kao H.A. (2015). A cyber-physical systems architecture for industry 4.0-based manufacturing systems. Manuf. Lett..

[7] Xu X., Lu Y., Vogel-Heuser B., Wang L. (2021). Industry 4.0 and Industry 5.0—Inception, conception and perception. J. Manuf. Syst..

[8] Ullah A.S. (2019). Fundamental Issues of Concept Mapping Relevant to Discipline-Based Education: A Perspective of Manufacturing Engineering. Educ. Sci..

[9] Doshi-Velez F., Kim B. (2017). Towards a rigorous science of interpretable machine learning. arXiv.

[10] Hassija V., Chamola V., Mahapatra A., Singal A., Goel D., Huang K., Scardapane S., Spinelli I., Mahmud M., Hussain A. (2024). Interpreting black-box models: A review on explainable artificial intelligence. Cogn. Comput..

[11] Greenberg M.R. (2014). Energy policy and research: The underappreciation of trust. Energy Res. Soc. Sci..

[12] Floridi L., Cowls J., Beltrametti M., Chatila R., Chazerand P., Dignum V., Luetge C., Madelin R., Pagallo U., Rossi F. (2018). AI4People—An ethical framework for a good AI society: Opportunities, risks, principles, and recommendations. Minds Mach..

[13] Veale M. (2020). A critical take on the policy recommendations of the EU high-level expert group on artificial intelligence. Eur. J. Risk Regul..

[14] AI N. (2023). Artificial Intelligence Risk Management Framework (AI RMF 1.0). https://nvlpubs.nist.gov/nistpubs/ai/nist.ai.100-1.pdf.

[15] Jobin A., Ienca M., Vayena E. (2019). The global landscape of AI ethics guidelines. Nat. Mach. Intell..

[16] Butt J. (2020). A Strategic Roadmap for the Manufacturing Industry to Implement Industry 4.0. Designs.

[17] Dixson-Declève S., Balland P.-A., Bria F., Charveriat C., Dunlop K., Giovannini E., Tataj D., Hidalgo C., Huang A., European Commission: DG Research and Innovation (2021). Industry 5.0, A Transformative Vision for Europe—Governing Systemic Transformations Towards a Sustainable Industry.

[18] Misra N.N., Dixit Y., Al-Mallahi A., Bhullar M.S., Upadhyay R., Martynenko A. (2022). IoT, Big Data, and Artificial Intelligence in Agriculture and Food Industry. IEEE Internet Things J..

[19] Zheng T., Marco Ardolino A.B., Perona M. (2021). The applications of Industry 4.0 technologies in manufacturing context: A systematic literature review. Int. J. Prod. Res..

[20] Angelopoulos A., Michailidis E.T., Nomikos N., Trakadas P., Hatziefremidis A., Voliotis S., Zahariadis T. (2020). Tackling Faults in the Industry 4.0 Era—A Survey of Machine-Learning Solutions and Key Aspects. Sensors.

[21] Müller J., European Commission: DG Research and Innovation (2020). Enabling Technologies for Industry 5.0—Results of a Workshop with Europe’s Technology Leaders.

[22] European Commission (2020). Skills Agenda for Europe: Promoting Skills and Talent in the European Union. https://research-and-innovation.ec.europa.eu/knowledge-publications-tools-and-data/publications/all-publications/enabling-technologies-industry-50_en.

[23] Claeys G., Tagliapietra S., Zachmann G. (2019). How to Make the European Green Deal Work.

[24] Puaschunder J.M. (2019). The legal and international situation of AI, robotics and big data with attention to healthcare. Report on Behalf of the European Parliament European Liberal Forum.

[25] Marcus J.S., Martens B., Carugati C., Bucher A., Godlovitch I., The European Health Data Space (2022). IPOL | Policy Department for Economic, Scientific and Quality of Life Policies, European Parliament Policy Department Studies. https://ssrn.com/abstract=4300393.

[26] Nikolinakos N.T. (2023). A European approach to excellence and trust: The 2020 white paper on artificial intelligence. EU Policy and Legal Framework for Artificial Intelligence, Robotics and Related Technologies—The AI Act.

[27] Ai H. (2019). High-level expert group on artificial intelligence. Ethics Guidel. Trust. AI.

[28] Mesarčík M., Solarova S., Podroužek J., Bieliková M. (2022). Stance on the proposal for a regulation laying down harmonised rules on artificial intelligence—artificial intelligence act. OSF Preprints.

[29] Cannarsa M. (2021). Ethics guidelines for trustworthy AI. The Cambridge Handbook of Lawyering in the Digital Age.

[30] Ryan M. (2023). The social and ethical impacts of artificial intelligence in agriculture: Mapping the agricultural AI literature. AI Soc..

[31] Wang J., Chuqiao X., Zhang J., Zhong R. (2021). Big data analytics for intelligent manufacturing systems: A review. J. Manuf. Syst..

[32] Nahavandi S. (2019). Industry 5.0—A human-centric solution. Sustainability.

[33] Rikap C. (2023). Same End by Different Means: Google, Amazon, Microsoft and Facebook’s Strategies to Dominate Artificial Intelligence. https://papers.ssrn.com/sol3/papers.cfm?abstract_id=4472222.

[34] Habib ur Rehman M., Dirir A., Salah K., Damiani E., Svetinovic D. (2021). TrustFed: A Framework for Fair and Trustworthy Cross-Device Federated Learning in IIoT. IEEE Trans. Ind. Inform..

[35] Vyhmeister E., Castane G., Östberg P.O., Thevenin S. (2022). A responsible AI framework: Pipeline contextualisation. AI Ethics.

[36] Vyhmeister E., Gonzalez-Castane G., Östberg P.O. (2022). Risk as a driver for AI framework development on manufacturing. AI Ethics.

[37] Wang Q., Liu D., Carmichael M., Aldini S., Lin C.T. (2022). Computational Model of Robot Trust in Human Co-Worker for Physical Human-Robot Collaboration. IEEE Robot. Autom. Lett..

[38] Wu J., Drew S., Dong F., Zhu Z., Zhou J. (2023). Topology-aware Federated Learning in Edge Computing: A Comprehensive Survey. arXiv.

[39] Reddy P., Pham V., B P., Deepa N., Dev K., Gadekallu T., Ruby R., Liyanage M. (2021). Industry 5.0: A Survey on Enabling Technologies and Potential Applications. J. Ind. Inf. Integr..

[40] Breque M., De Nul L., Petridis A., Directorate-General for Research and Innovation (European Commission) (2021). Industry 5.0: Towards a Sustainable, Human-Centric and Resilient European Industry.

[41] Fahle S., Prinz C., Kuhlenkötter B. (2020). Systematic review on machine learning (ML) methods for manufacturing processes—Identifying artificial intelligence (AI) methods for field application. Procedia CIRP.

[42] IBM How Is AI Being Used in Manufacturing? AI in Manufacturing. https://www.ibm.com/think/topics/ai-in-manufacturing.

[43] Zonta T., da Costa C.A., da Rosa Righi R., de Lima M.J., da Trindade E.S., Li G.P. (2020). Predictive maintenance in the Industry 4.0: A systematic literature review. Comput. Ind. Eng..

[44] Qarout Y., Begg M., Fearon L., Russell D., Pietrow N., Rahman M., McLeod S., Chakravorti N., Winter T., Fortune J. (2024). Trustworthy AI Framework: A Comprehensive Review of AI Standards Policies and a Practical Guideline to Their Application in Manufacturing. https://www.the-mtc.org/api/sites/default/files/2024-07/Trustworthy%20AI%20Framework.pdf.

[45] European Commission, High-Level Expert Group on AI (2019). Ethics Guidelines for Trustworthy AI. https://digital-strategy.ec.europa.eu/en/library/ethics-guidelines-trustworthy-ai.

[46] Reinhardt K. (2023). Trust and trustworthiness in AI ethics. AI Ethics.

[47] Idamia S., Benseddik H. (2024). Advancing Industry 5.0: An Extensive Review of AI Integration. Industry 5.0 and Emerging Technologies: Transformation Through Technology and Innovations.

[48] Dimitrakopoulos G., Varga P., Gutt T., Schneider G., Ehm H., Hoess A., Tauber M., Karathanasopoulou K., Lackner A., Delsing J. (2024). Industry 5.0: Research Areas and Challenges With Artificial Intelligence and Human Acceptance. IEEE Ind. Electron. Mag..

[49] Keller N. (2013). Cybersecurity Framework. https://www.nist.gov/cyberframework.

[50] International Organization for Standardization (2022). Information Security, Cybersecurity and Privacy Protection—Information Security Management Systems—Requirements.

[51] Wachter S., Mittelstadt B., Floridi L. (2017). Why a right to explanation of automated decision-making does not exist in the general data protection regulation. Int. Data Priv. Law.

[52] Ala-Pietilä P., Bonnet Y., Bergmann U., Bielikova M., Bonefeld-Dahl C., Bauer W., Bouarfa L., Chatila R., Coeckelbergh M., Dignum V. (2020). The Assessment List for Trustworthy Artificial Intelligence (ALTAI).

[53] Martini B., Bellisario D., Coletti P. (2024). Human-Centered and Sustainable Artificial Intelligence in Industry 5.0: Challenges and Perspectives. Sustainability.

[54] Yusuf S.O., Durodola R.L., Ocran G., Abubakar J.E., Echere A.Z., Paul-Adeleye A.H. (2024). Challenges and opportunities in AI and digital transformation for SMEs: A cross-continental perspective. World J. Adv. Res. Rev..

[55] Wilson H.J., Daugherty P.R. (2018). Collaborative intelligence: Humans and AI are joining forces. Harv. Bus. Rev..

[56] Thomas M. Dangerous Risks of Artificial Intelligence. Volume 6. https://builtin.com/artificial-intelligence/risks-of-artificial-intelligence.

[57] Angwin J., Larson J., Mattu S., Kirchner L. (2022). Machine bias. Ethics of Data and Analytics.

[58] Zhang M. (2015). Google Photos Tags Two African-Americans as Gorillas Through Facial Recognition Software. https://www.forbes.com/sites/mzhang/2015/07/01/google-photos-tags-two-african-americans-as-gorillas-through-facial-recognition-software/.

[59] Dastin J. (2022). Amazon scraps secret AI recruiting tool that showed bias against women. Ethics of Data and Analytics.

[60] Kohli P., Chadha A. (2019). Enabling pedestrian safety using computer vision techniques: A case study of the 2018 uber Inc. self-driving car crash. Proceedings of the Future of Information and Communication Conference.

[61] Fan W., Liu J., Zhu S., Pardalos P.M. (2020). Investigating the impacting factors for the healthcare professionals to adopt artificial intelligence-based medical diagnosis support system (AIMDSS). Ann. Oper. Res..

[62] Elliott A. (2019). The Culture of AI: Everyday Life and the Digital Revolution.

[63] Marcus G., Davis E. (2019). Rebooting AI: Building Artificial Intelligence We Can Trust.

[64] Floridi L., Cowls J. (2022). A unified framework of five principles for AI in society. Machine Learning and the City: Applications in Architecture and Urban Design.

[65] Floridi L., Cowls J., King T.C., Taddeo M. (2021). How to design AI for social good: Seven essential factors. Ethics, Governance, and Policies in Artificial Intelligence.

[66] Pichai S. (2018). AI at Google: Our principles. Keyword.

[67] UNI Global Union Top 10 principles for ethical artificial intelligence. The FutureWorld ofWork, UNI Global Union: Nyon, Switzerland, 2017. https://uniglobalunion.org/report/10-principles-for-ethical-artificial-intelligence/.

[68] Zeng Y., Lu E., Huangfu C. (2018). Linking artificial intelligence principles. arXiv.

[69] Hagendorff T. (2020). The ethics of AI ethics: An evaluation of guidelines. Minds Mach..

[70] Kaur D., Uslu S., Durresi A. (2021). Requirements for trustworthy artificial intelligence–a review. Proceedings of the Advances in Networked-Based Information Systems: The 23rd International Conference on Network-Based Information Systems (NBiS-2020) 23.

[71] Kumar A., Braud T., Tarkoma S., Hui P. Trustworthy AI in the age of pervasive computing and big data. Proceedings of the 2020 IEEE International Conference on Pervasive Computing and Communications Workshops (PerCom Workshops).

[72] Smuha N.A. (2019). The EU approach to ethics guidelines for trustworthy artificial intelligence. Comput. Law Rev. Int..

[73] Daugherty P.R., Wilson H.J. (2018). Human+ Machine: Reimagining Work in the Age of AI.

[74] Goodman B., Flaxman S. (2017). European Union regulations on algorithmic decision-making and a “right to explanation”. AI Mag..

[75] Dignum V. (2018). Responsible Artificial Intelligence: Designing AI for Human Values. ITU J. ICT Discov..

[76] Shin D., Park Y.J. (2019). Role of fairness, accountability, and transparency in algorithmic affordance. Comput. Hum. Behav..

[77] Flores A.W., Bechtel K., Lowenkamp C.T. (2016). False positives, false negatives, and false analyses: A rejoinder to machine bias: There’s software used across the country to predict future criminals. and it’s biased against blacks. Fed. Probat..

[78] Wieringa M. What to account for when accounting for algorithms: A systematic literature review on algorithmic accountability. Proceedings of the 2020 Conference on Fairness, Accountability, and Transparency.

[79] Islam M.R., Ahmed M.U., Barua S., Begum S. (2022). A systematic review of explainable artificial intelligence in terms of different application domains and tasks. Appl. Sci..

[80] Chou Y.L., Moreira C., Bruza P., Ouyang C., Jorge J. (2022). Counterfactuals and causability in explainable artificial intelligence: Theory, algorithms, and applications. Inf. Fusion.

[81] Ahmed I., Jeon G., Piccialli F. (2022). From artificial intelligence to explainable artificial intelligence in industry 4.0: A survey on what, how, and where. IEEE Trans. Ind. Inform..

[82] Khosravi H., Shum S.B., Chen G., Conati C., Tsai Y.S., Kay J., Knight S., Martinez-Maldonado R., Sadiq S., Gašević D. (2022). Explainable artificial intelligence in education. Comput. Educ. Artif. Intell..

[83] Rawal A., McCoy J., Rawat D.B., Sadler B.M., Amant R.S. (2021). Recent advances in trustworthy explainable artificial intelligence: Status, challenges, and perspectives. IEEE Trans. Artif. Intell..

[84] Albahri A.S., Duhaim A.M., Fadhel M.A., Alnoor A., Baqer N.S., Alzubaidi L., Albahri O.S., Alamoodi A.H., Bai J., Salhi A. (2023). A systematic review of trustworthy and explainable artificial intelligence in healthcare: Assessment of quality, bias risk, and data fusion. Inf. Fusion.

[85] Albahri A., Al-Qaysi Z., Alzubaidi L., Alnoor A., Albahri O., Alamoodi A., Bakar A.A. (2023). A Systematic Review of Using Deep Learning Technology in the Steady-State Visually Evoked Potential-Based Brain-Computer Interface Applications: Current Trends and Future Trust Methodology. Int. J. Telemed. Appl..

[86] Velmurugan M., Ouyang C., Moreira C., Sindhgatta R. (2021). Evaluating stability of post-hoc explanations for business process predictions. Proceedings of the International Conference on Service-Oriented Computing.

[87] Selbst A., Powles J. “Meaningful information” and the right to explanation. Proceedings of the Conference on Fairness, Accountability and Transparency.

[88] Velmurugan M., Ouyang C., Moreira C., Sindhgatta R. (2021). Evaluating fidelity of explainable methods for predictive process analytics. Proceedings of the International Conference on Advanced Information Systems Engineering.

[89] Sreedharan S., Srivastava S., Kambhampati S. (2021). Using state abstractions to compute personalized contrastive explanations for AI agent behavior. Artif. Intell..

[90] Shin D. (2021). The effects of explainability and causability on perception, trust, and acceptance: Implications for explainable AI. Int. J. Hum.-Comput. Stud..

[91] Lim W.X., Chen Z., Ahmed A. (2022). The adoption of deep learning interpretability techniques on diabetic retinopathy analysis: A review. Med Biol. Eng. Comput..

[92] Huang Y., Chen D., Zhao W., Lv Y., Wang S. (2022). Deep patch learning algorithms with high interpretability for regression problems. Int. J. Intell. Syst..

[93] Yang C., Rangarajan A., Ranka S. Global model interpretation via recursive partitioning. Proceedings of the 2018 IEEE 20th International Conference on High Performance Computing and Communications; IEEE 16th International Conference on Smart City; IEEE 4th International Conference on Data Science and Systems (HPCC/SmartCity/DSS).

[94] Moreira C., Chou Y.L., Velmurugan M., Ouyang C., Sindhgatta R., Bruza P. (2021). LINDA-BN: An interpretable probabilistic approach for demystifying black-box predictive models. Decis. Support Syst..

[95] Lyu D., Yang F., Kwon H., Dong W., Yilmaz L., Liu B. (2021). Tdm: Trustworthy decision-making via interpretability enhancement. IEEE Trans. Emerg. Top. Comput. Intell..

[96] Reed C. (2018). How should we regulate artificial intelligence?. Philos. Trans. R. Soc. A: Math. Phys. Eng. Sci..

[97] Wickramanayake B., He Z., Ouyang C., Moreira C., Xu Y., Sindhgatta R. (2022). Building interpretable models for business process prediction using shared and specialised attention mechanisms. Knowl.-Based Syst..

[98] Thiebes S., Lins S., Sunyaev A. (2021). Trustworthy artificial intelligence. Electron. Mark..

[99] Sindhgatta R., Ouyang C., Moreira C. (2020). Exploring interpretability for predictive process analytics. Proceedings of the International Conference on Service-Oriented Computing.

[100] Bhatt U., Xiang A., Sharma S., Weller A., Taly A., Jia Y., Ghosh J., Puri R., Moura J.M., Eckersley P. Explainable machine learning in deployment. Proceedings of the 2020 Conference on Fairness, Accountability, and Transparency.

[101] IBM (2025). AI Explainability 360. https://research.ibm.com/blog/ai-explainability-360.

[102] Ribeiro M.T., Singh S., Guestrin C. (2016). Why should I trust you? Explaining the predictions of any classifier. Proceedings of the 22nd ACM SIGKDD International Conference on Knowledge Discovery and Data Mining.

[103] Lundberg S.M., Lee S.I. (2017). A unified approach to interpreting model predictions. Advances in Neural Information Processing Systems.

[104] Chen J., Kanan C., Fowlkes C.C. XAITK Saliency: An Open Source Explainable AI Toolkit for Visual Saliency. https://ojs.aaai.org/index.php/AAAI/article/view/26871.

[105] Yin H., Xie K., Zhou Y., Liu J., Zhang Z., Liu P., Wang W., Zhao Y. (2022). Fidelity in Explanation Methods: A Comprehensive Study. arXiv.

[106] Ieracitano C., Mammone N., Hussain A., Morabito F.C. (2022). A novel explainable machine learning approach for EEG-based brain-computer interface systems. Neural Comput. Appl..

[107] Ras G., Xie N., Van Gerven M., Doran D. (2022). Explainable deep learning: A field guide for the uninitiated. J. Artif. Intell. Res..

[108] De Waal A., Joubert J.W. (2022). Explainable Bayesian networks applied to transport vulnerability. Expert Syst. Appl..

[109] Mao C., Lin R., Towey D., Wang W., Chen J., He Q. (2021). Trustworthiness prediction of cloud services based on selective neural network ensemble learning. Expert Syst. Appl..

[110] Srinivasan R., González B.S.M. (2022). The role of empathy for artificial intelligence accountability. J. Responsible Technol..

[111] Choudhury A., Asan O. (2022). Impact of accountability, training, and human factors on the use of artificial intelligence in healthcare: Exploring the perceptions of healthcare practitioners in the US. Hum. Factors Healthc..

[112] Fong S., Dey N., Joshi A. ICT analysis and applications. Proceedings of the ICT4SD.

[113] Cruz B.S., de Oliveira Dias M. (2020). Crashed boeing 737-max: Fatalities or malpractice. GSJ.

[114] Schwartz R., Schwartz R., Vassilev A., Greene K., Perine L., Burt A., Hall P. (2022). Towards a Standard for Identifying and Managing Bias in Artificial Intelligence.

[115] Gevaert C.M., Carman M., Rosman B., Georgiadou Y., Soden R. (2021). Fairness and accountability of AI in disaster risk management: Opportunities and challenges. Patterns.

[116] Königstorfer F., Thalmann S. (2022). AI Documentation: A path to accountability. J. Responsible Technol..

[117] Rahwan I., Cebrian M., Obradovich N., Bongard J., Bonnefon J.F., Breazeal C., Crandall J.W., Christakis N.A., Couzin I.D., Jackson M.O. (2019). Machine behaviour. Nature.

[118] Morley J., Floridi L., Kinsey L., Elhalal A. (2020). From what to how: An initial review of publicly available AI ethics tools, methods and research to translate principles into practices. Sci. Eng. Ethics.

[119] Guidotti R., Monreale A., Ruggieri S., Turini F., Giannotti F., Pedreschi D. (2018). A survey of methods for explaining black box models. ACM Comput. Surv. (CSUR).

[120] Omeiza D., Web H., Jirotka M., Kunze L. Towards accountability: Providing intelligible explanations in autonomous driving. Proceedings of the 2021 IEEE Intelligent Vehicles Symposium (IV).

[121] Kadambi A. (2021). Achieving fairness in medical devices. Science.

[122] Mehrabi N., Morstatter F., Saxena N., Lerman K., Galstyan A. (2021). A survey on bias and fairness in machine learning. ACM Comput. Surv. (CSUR).

[123] Madaio M., Egede L., Subramonyam H., Wortman Vaughan J., Wallach H. Assessing the fairness of ai systems: Ai practitioners’ processes, challenges, and needs for support. Proceedings of the ACM on Human-Computer Interaction.

[124] Rudin C. (2019). Stop explaining black box machine learning models for high stakes decisions and use interpretable models instead. Nat. Mach. Intell..

[125] von Zahn M., Feuerriegel S., Kuehl N. (2022). The cost of fairness in AI: Evidence from e-commerce. Bus. Inf. Syst. Eng..

[126] Pastaltzidis I., Dimitriou N., Quezada-Tavarez K., Aidinlis S., Marquenie T., Gurzawska A., Tzovaras D. Data augmentation for fairness-aware machine learning: Preventing algorithmic bias in law enforcement systems. Proceedings of the 2022 ACM Conference on Fairness, Accountability, and Transparency.

[127] Chouldechova A., Benavides-Prado D., Fialko O., Vaithianathan R. A case study of algorithm-assisted decision making in child maltreatment hotline screening decisions. Proceedings of the Conference on Fairness, Accountability and Transparency.

[128] Dwork C., Hardt M., Pitassi T., Reingold O., Zemel R. Fairness through awareness. Proceedings of the 3rd Innovations in Theoretical Computer Science Conference.

[129] Oneto L., Chiappa S. (2020). Recent Trends in Learning from Data.

[130] Kusner M.J., Loftus J., Russell C., Silva R. (2017). Counterfactual fairness. Adv. Neural Inf. Process. Syst..

[131] Grari V., Lamprier S., Detyniecki M. (2023). Adversarial learning for counterfactual fairness. Mach. Learn..

[132] Santos F.P., Santos F.C., Paiva A., Pacheco J.M. (2015). Evolutionary dynamics of group fairness. J. Theor. Biol..

[133] Khalili M.M., Zhang X., Abroshan M. (2021). Fair sequential selection using supervised learning models. Adv. Neural Inf. Process. Syst..

[134] Zheng Y., Wang S., Zhao J. (2021). Equality of opportunity in travel behavior prediction with deep neural networks and discrete choice models. Transp. Res. Part C Emerg. Technol..

[135] Besse P., del Barrio E., Gordaliza P., Loubes J.M., Risser L. (2022). A survey of bias in machine learning through the prism of statistical parity. Am. Stat..

[136] Feuerriegel S., Dolata M., Schwabe G. (2020). Fair AI: Challenges and opportunities. Bus. Inf. Syst. Eng..

[137] Chen Y., Huerta E., Duarte J., Harris P., Katz D.S., Neubauer M.S., Diaz D., Mokhtar F., Kansal R., Park S.E. (2022). A FAIR and AI-ready Higgs boson decay dataset. Sci. Data.

[138] AI Fairness 360 (2025). AI Fairness 360. https://research.ibm.com/blog/ai-fairness-360.

[139] Fairlearn (2025). Fairlearn. https://fairlearn.org/.

[140] What-If Tool (2025). What-If Tool. https://pair-code.github.io/what-if-tool/.

[141] Aequitas (2025). Aequitas. https://arxiv.org/abs/1811.05577.

[142] Google Research (2019). ML Fairness Gym: A Tool for Exploring Long-Term Impacts of Machine Learning Systems. https://research.google/blog/ml-fairness-gym-a-tool-for-exploring-long-term-impacts-of-machine-learning-systems/.

[143] AI Now Institute (2022). Algorithmic Impact Assessments: A Practical Framework for Public Agency Accountability. https://ainowinstitute.org/publications/algorithmic-impact-assessments-report-2.

[144] UK Government (2020). Data Ethics Framework 2020. https://assets.publishing.service.gov.uk/media/5f74a4958fa8f5188dad0e99/Data_Ethics_Framework_2020.pdf.

[145] Swiss Re (2023). A Journey into Responsible AI. https://www.swissre.com/institute/.

[146] Hauer M.P., Adler R., Zweig K. Assuring fairness of algorithmic decision making. Proceedings of the 2021 IEEE International Conference on Software Testing, Verification and Validation Workshops (ICSTW), Porto de Galinhas.

[147] Amodei D., Olah C., Steinhardt J., Christiano P., Schulman J., Mané D. (2016). Concrete problems in AI safety. arXiv.

[148] Tan X., Xu K., Cao Y., Zhang Y., Ma L., Lau R.W. (2021). Night-time scene parsing with a large real dataset. IEEE Trans. Image Process..

[149] Zhang M., Zhang Y., Zhang L., Liu C., Khurshid S. Deeproad: Gan-based metamorphic testing and input validation framework for autonomous driving systems. Proceedings of the 33rd ACM/IEEE International Conference on Automated Software Engineering.

[150] Akhtar N., Mian A. (2018). Threat of adversarial attacks on deep learning in computer vision: A survey. IEEE Access.

[151] Chakraborty A., Alam M., Dey V., Chattopadhyay A., Mukhopadhyay D. (2018). Adversarial attacks and defences: A survey. arXiv.

[152] Li Y., Jiang Y., Li Z., Xia S.T. (2022). Backdoor learning: A survey. IEEE Trans. Neural Netw. Learn. Syst..

[153] Silva S.H., Najafirad P. (2020). Opportunities and challenges in deep learning adversarial robustness: A survey. arXiv.

[154] Yuan X., He P., Zhu Q., Li X. (2019). Adversarial examples: Attacks and defenses for deep learning. IEEE Trans. Neural Netw. Learn. Syst..

[155] Vorobeychik Y., Kantarcioglu M. (2018). Adversarial Machine Learning.

[156] Tong L., Li B., Hajaj C., Xiao C., Zhang N., Vorobeychik Y. Improving robustness of {ML} classifiers against realizable evasion attacks using conserved features. Proceedings of the 28th USENIX Security Symposium (USENIX Security 19).

[157] Wu T., Tong L., Vorobeychik Y. (2019). Defending against physically realizable attacks on image classification. arXiv.

[158] Tramèr F., Zhang F., Juels A., Reiter M.K., Ristenpart T. Stealing machine learning models via prediction {APIs}. Proceedings of the 25th USENIX security symposium (USENIX Security 16).

[159] Ramachandra R., Busch C. (2017). Presentation attack detection methods for face recognition systems: A comprehensive survey. ACM Comput. Surv. (CSUR).

[160] Machado G.R., Silva E., Goldschmidt R.R. (2021). Adversarial machine learning in image classification: A survey toward the defender’s perspective. ACM Comput. Surv. (CSUR).

[161] Exforsys (2011). What Is Monkey Testing. http://www.exforsys.com/tutorials/testing-types/monkey-testing.html.

[162] Ma L., Juefei-Xu F., Zhang F., Sun J., Xue M., Li B., Chen C., Su T., Li L., Liu Y. Deepgauge: Multi-granularity testing criteria for deep learning systems. Proceedings of the 33rd ACM/IEEE International Conference on Automated Software Engineering.

[163] Pei K., Cao Y., Yang J., Jana S. Deepxplore: Automated whitebox testing of deep learning systems. Proceedings of the 26th Symposium on Operating Systems Principles.

[164] Carlini N., Wagner D. Towards evaluating the robustness of neural networks. Proceedings of the 2017 IEEE Symposium on Security and Privacy (SP).

[165] Su D., Zhang H., Chen H., Yi J., Chen P.Y., Gao Y. Is robustness the cost of accuracy?–a comprehensive study on the robustness of 18 deep image classification models. Proceedings of the European Conference on Computer Vision (ECCV).

[166] Boopathy A., Weng T.W., Chen P.Y., Liu S., Daniel L. Cnn-cert: An efficient framework for certifying robustness of convolutional neural networks. Proceedings of the AAAI Conference on Artificial Intelligence.

[167] Zhang H., Weng T.W., Chen P.Y., Hsieh C.J., Daniel L. (2018). Efficient neural network robustness certification with general activation functions. Adv. Neural Inf. Process. Syst..

[168] Nicolae M.I., Sinn M., Tran M.N., Buesser B., Rawat A., Wistuba M., Zantedeschi V., Baracaldo N., Chen B., Ludwig H. (2018). Adversarial Robustness Toolbox v1.0.0. arXiv.

[169] Papernot N., Faghri F., Carlini N., Goodfellow I., Feinman R., Kurakin A., Xie C., Sharma Y., Brown T., Roy A. (2018). Technical Report on the CleverHans v2.1.0 Adversarial Examples Library. arXiv.

[170] Rauber J., Brendel W., Bethge M. (2018). Foolbox: A Python toolbox to benchmark the robustness of machine learning models. arXiv.

[171] Soklaski R., Goodwin J., Brown O., Yee M., Matterer J. (2022). Tools and Practices for Responsible AI Engineering. arXiv.

[172] Li Y., Jin W., Xu H., Tang J. (2020). DeepRobust: A PyTorch Library for Adversarial Attacks and Defenses. arXiv.

[173] Chen J., Su Y., Song Z., Wang B., Tang R., Zhao S., Yang Y., Zhang J., Kumar M., Xu H. (2019). Model-agnostic meta-learning for fast adaptation of deep networks. arXiv.

[174] Radford A., Kim J., Hallacy C., Ramesh A., Goh G., Agarwal K., Clark J., Krueger G., Chen M., Sutskever I. (2021). Learning Transferable Visual Models From Natural Language Supervision. arXiv.

[175] Brown T.B., Mann B., Ryder N., Subbiah M., Kaplan J., Dhariwal P., Neelakantan A., Shinn E., Ibarz J., Wu L. (2019). Language Models are Unsupervised Multitask Learners. arXiv.

[176] Liu Z., Lin Y., Cao Y., Zhang F., Zhang Z., Wang X., Zhang T., Chen X., Zeng R., Yu L. (2022). Data-efficient image transformers for downstream tasks. arXiv.

[177] Yu H., Zheng F., Hu H., Gu S., Zhang S., Li H. (2020). Learning to Learn with Conditional Class Dependencies. arXiv.

[178] He M., Li Z., Liu C., Shi D., Tan Z. (2020). Deployment of Artificial Intelligence in Real-World Practice: Opportunity and Challenge. Asia-Pac. J. Ophthalmol..

[179] Peres R.S., Jia X., Lee J., Sun K., Colombo A.W., Barata J. (2020). Industrial artificial intelligence in industry 4.0-systematic review, challenges and outlook. IEEE Access.

[180] Ahsan M., Hon S.T., Albarbar A. (2020). Development of Novel Big Data Analytics Framework for Smart Clothing. IEEE Access.

[181] Wang P., Wang K., Wang D., Liu H. (2024). The Impact of Manufacturing Transformation in Digital Economy Under Artificial Intelligence. IEEE Access.

[182] Colombi L., Gilli A., Dahdal S., Boleac I., Tortonesi M., Stefanelli C., Vignoli M. A Machine Learning Operations Platform for Streamlined Model Serving in Industry 5.0. Proceedings of the NOMS 2024-2024 IEEE Network Operations and Management Symposium.

[183] Yu W., Liu Y., Dillon T., Rahayu W., Mostafa F. (2022). An Integrated Framework for Health State Monitoring in a Smart Factory Employing IoT and Big Data Techniques. IEEE Internet Things J..

[184] Hariharakrishnan J., Mohanavalli S., Srividya, Sundhara Kumar K.B. Survey of pre-processing techniques for mining big data. Proceedings of the 2017 International Conference on Computer, Communication and Signal Processing (ICCCSP).

[185] Shahriar S., Allana S., Hazratifard S.M., Dara R. (2023). A Survey of Privacy Risks and Mitigation Strategies in the Artificial Intelligence Life Cycle. IEEE Access.

[186] Kemnitz J., Weissenfeld A., Schoeffl L., Stiftinger A., Rechberger D., Prangl B., Kaufmann T., Hiessl T., Holly S., Heistracher C. An Edge Deployment Framework to Scale AI in Industrial Applications. Proceedings of the 2023 IEEE 7th International Conference on Fog and Edge Computing (ICFEC).

